# *Cannabis* labelling is associated with genetic variation in terpene synthase genes

**DOI:** 10.1038/s41477-021-01003-y

**Published:** 2021-10-14

**Authors:** Sophie Watts, Michel McElroy, Zoë Migicovsky, Hugo Maassen, Robin van Velzen, Sean Myles

**Affiliations:** 1 Department of Plant, Food and Environmental Sciences, Dalhousie University, Truro, Nova Scotia Canada; 2 Bedrocan International, Veendam, the Netherlands; 3 Biosystematics Group, Wageningen University, Wageningen, the Netherlands

**Keywords:** Genomics, Natural variation in plants

## Abstract

Analysis of over 100 *Cannabis* samples quantified for terpene and cannabinoid content and genotyped for over 100,000 single nucleotide polymorphisms indicated that Sativa- and Indica-labelled samples were genetically indistinct on a genome-wide scale. Instead, we found that *Cannabis* labelling was associated with variation in a small number of terpenes whose concentrations are controlled by genetic variation at tandem arrays of terpene synthase genes.

## Abstract (web summary)

By quantifying over 100 *Cannabis* samples for terpene and cannabinoid content and genotyping them for over 100,000 single nucleotide polymorphisms, this study finds that *Cannabis* labelling is associated with genetic variants in terpene synthase genes.

## Main

*Cannabis* has been consumed for its psychoactive properties for over 2,500 years, and its estimated global market value is US$340 billion^1–3^. Because it is a widely used drug that is increasingly being legalized for medicinal and recreational use, it is critical that *Cannabis*’s genetic and chemical variation be accurately quantified and communicated. The vernacular labels Sativa and Indica (not to be confused with the taxonomic names *C. sativa sativa* L. and *C. sativa indica* Lam.) are routinely assigned to *Cannabis* cultivars by breeders, retailers and users to describe a cultivar’s morphology, aromas and/or psychoactive effects^4^. However, it is unclear whether these labels capture meaningful information about *Cannabis* genetic and chemical variation.

*Cannabis* genomics research has thus far largely focused on the characterization of genes underlying the production of the cannabinoids cannabidiol (CBD) and tetrahydrocannabinol (THC)^5–8^. However, *Cannabis* produces hundreds of aromatic terpenes that drive consumer preference and are frequently associated with Sativa and Indica labels^4,9^. In addition, there is evidence to suggest that a cultivar’s terpene profile affects its psychoactive properties^10,11^. To date, various terpene synthase genes have been identified in *Cannabis*; however, the genetic control of terpene variation across *Cannabis* cultivars remains largely unexplored^12–15^.

**Extended Data Fig. 1:**
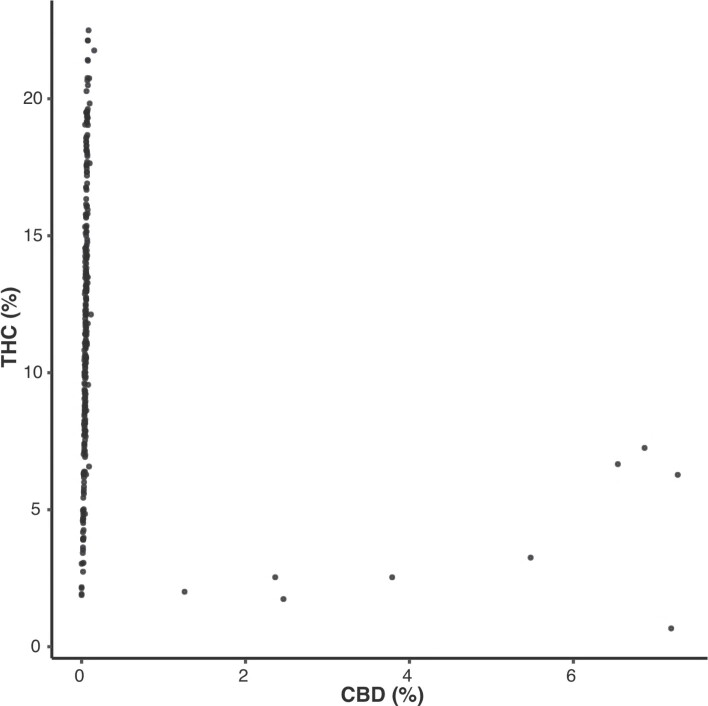
CBD vs THC content. Plot of percent CBD versus percent THC content.

Here we re-analysed 297 samples of drug-type *Cannabis* that were previously quantified for 40 terpenes and cannabinoids using gas chromatography–mass spectrometry (GC–MS)^16^ (Supplementary Table 1 and Extended Data Fig. 1), and we paired these data with 116,296 newly generated single nucleotide polymorphisms (SNPs) from 137 of these samples from which sufficient high-quality DNA could be extracted. We determined the degree to which the genomic and GC–MS data corresponded to a five-point labelling scale ranging from 1 (100% Sativa) to 5 (100% Indica) as reported by sample sources.

**Fig. 1:**
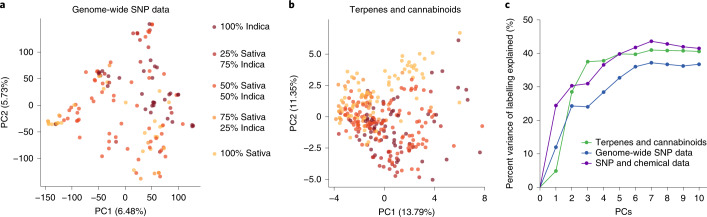
PCA. **a**, Genome-wide SNP data. **b**, Terpenes and cannabinoids. Each dot represents a *Cannabis* sample and is coloured by the labelling scale ranging from 100% Sativa to 100% Indica. **c**, The percent variance explained by PCs from the genome-wide SNP data (blue), from the terpene and cannabinoid data (green) and from both the genetic and chemical data (purple). The *y* axis shows the percent variance explained as PCs are added to linear models where the Sativa–Indica labelling scale is the dependent variable.

**Extended Data Fig. 2:**
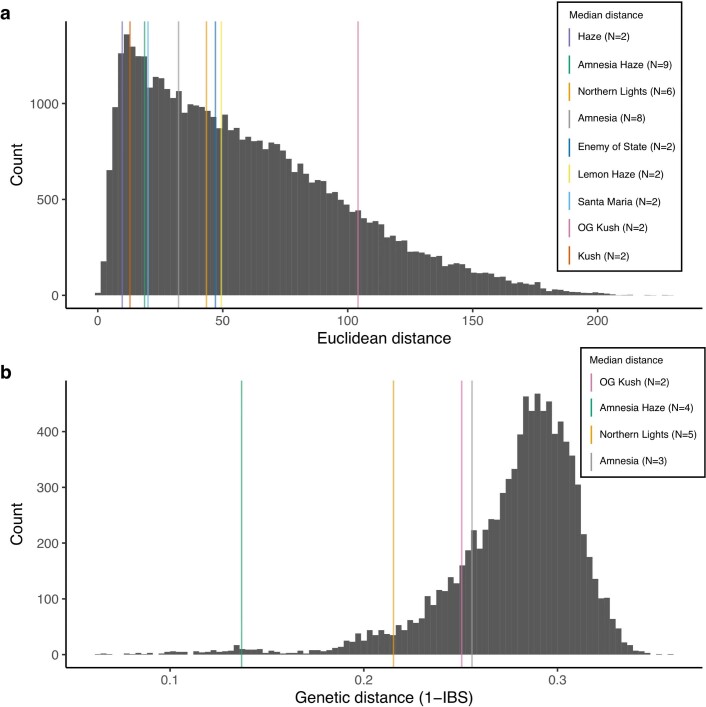
Pairwise chemical and genetic distances. Histograms of **a**) pairwise chemical distances and **b**) pairwise genetic distances among all pairs of samples. Vertical lines indicate the median distance between pairs of samples with the same name.

Principal component analysis (PCA) of the genomic data showed no clear clustering according to sample labels (Fig. 1a). Even though PC1 and PC2 were significantly correlated with the Sativa–Indica scale, the variance explained by the primary PCs was low (PC1: *R*^2^ = 0.12, *P* = 2.1 × 10^−5^; PC2: *R*^2^ = 0.12, *P* = 1.8 × 10^−5^). Furthermore, the overall genetic structure (captured by including the first ten PCs of the genomic data in a linear model) explained only 37% of the variance in labelling (Fig. 1c). Sativa–Indica labels thus do not accurately reflect genetic relatedness, which is consistent with previous work^17,18^. In addition, we determined that pairs of samples with identical cultivar names (for example, OG Kush) were often as genetically and chemically distant from each other as pairs of samples with different names (Extended Data Fig. 2). This is consistent with previous studies indicating that cultivar names were not reliable indicators of a sample’s genetic or chemical identity^17,19–21^.

**Extended Data Fig. 3:**
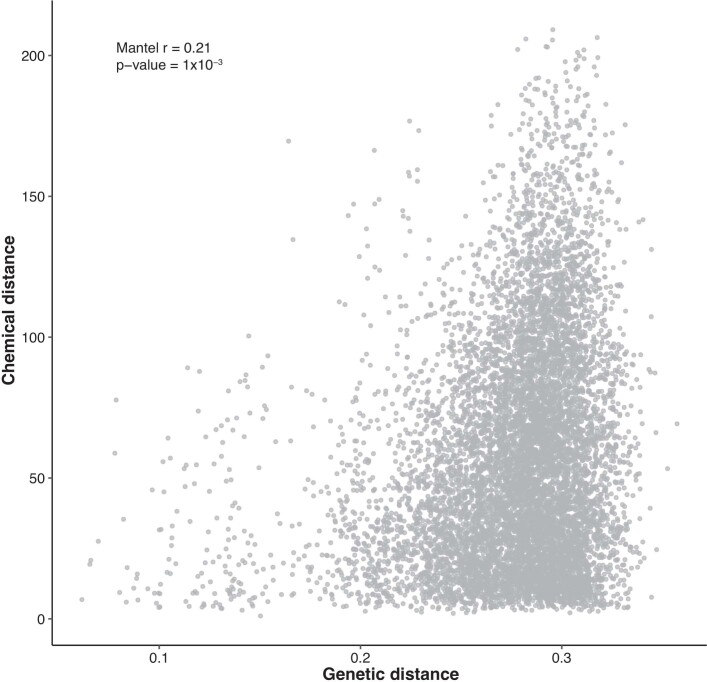
Correlation of chemical and genetic pairwise distances. Plot of genetic distance versus chemical distance between pairs of samples. The Mantel r statistic and p-value are reported.

Similar to the PCA of the genome-wide SNP data, the PCA of the terpene and cannabinoid profiles provided poor separation of samples according to their Sativa–Indica labels (Fig. 1b). Nevertheless, we observed significant correlations between the first two PCs and the Sativa–Indica scale (PC1: *R*^2^ = 0.049, *P* = 7.5 × 10^−5^; PC2: *R*^2^ = 0.24, *P* = 3.7 × 10^−19^). Including the first ten PCs from the terpene and cannabinoid profiles in a linear model accounted for only 41% of the variance in labelling (Fig. 1c). The pairwise genetic and chemical relatedness matrices were correlated (Mantel *r* = 0.21, *P* = 1 × 10^−3^, Extended Data Fig. 3), and a linear model including the first ten PCs from both the genomic and chemical profiles captured only 41% (Fig. 1c; *P* = 3.1 × 10^−10^) of the variance in labelling. Since the overall patterns of genetic and chemical relatedness could not fully account for the labels applied to *Cannabis* samples, we aimed to determine which individual chemicals were the strongest predictors of Sativa–Indica labelling.

**Fig. 2:**
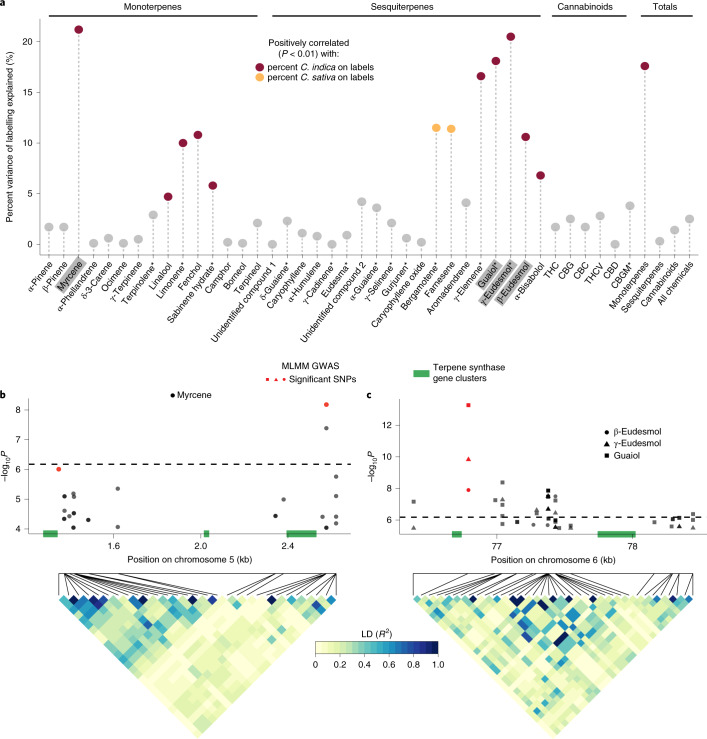
The genetic control of terpenes underlying *Cannabis* labelling. **a**, The percent variance of the five-point Sativa–Indica labelling scale that is explained by terpene and cannabinoid concentrations from Pearson correlations. The *P* values were Bonferroni-adjusted for multiple comparisons. The asterisks denote chemicals with tentative identifications. GWAS results are shown for chemicals highlighted in grey. **b**,**c**, Manhattan plots of mixed linear model (MLM) GWAS for myrcene on chromosome 5 (**b**) and for guaiol, γ-eudesmol and β-eudesmol on chromosome 6 (**c**). The significance thresholds from the MLM are shown as horizontal dashed lines. Significant SNPs from the MLMM GWAS are red. Terpene synthase gene clusters are green. Below the Manhattan plots are heat maps of the pairwise LD (*R*^2^) between pairs of SNPs that appear in the Manhattan plots.

Of the 40 measured terpenes and cannabinoids, 12 (30%) were correlated with the Sativa–Indica scale at *P* < 0.01 (Fig. 2a and Supplementary Fig. 1). Sativa content was positively correlated with the concentrations of bergamotene (*R*^2^ = 0.12, *P* = 9.26 × 10^−8^) and farnesene (*R*^2^ = 0.11, *P* = 1.09 × 10^−7^), which impart tea-like and fruity aromas, respectively^22,23^. This is consistent with descriptions of Sativa cultivars as having a ‘sweet’ or ‘herbal’ aroma^4,9^. The strongest correlation was between Indica content and myrcene, whose concentration explained 21.2% of the variation in labelling (*P* = 2.29 × 10^−15^; Fig. 2a). The sedative effect and earthy aroma attributed to high myrcene content are often reported by recreational users to be characteristic of Indica cultivars^10,24–26^. We also observed significant positive correlations between Indica labelling and three sesquiterpenes: guaiol (*R*^2^ = 0.18, *P* = 7.7 × 10^−13^), γ-eudesmol (*R*^2^ = 0.11, *P* = 3.8 × 10^−7^) and β-eudesmol (*R*^2^ = 0.21, *P* = 8.2 × 10^−15^). Hillig^27^ found that these three sesquiterpenes were associated with plants from Afghanistan, which is considered the region of origin for Indica cultivars.

Previous chemical analyses of *Cannabis* have suggested that the distinction between Sativa and Indica is best explained by differences in the concentrations of specific monoterpenes and sesquiterpenes^19,28–30^. In addition, the contrasting aromas that have been associated with Sativa (that is, sweet) and Indica (that is, earthy) were key discriminators in a sensory evaluation of *Cannabis* cultivars and mediated customers’ perceptions of potency and quality^9^. As a previous study suggested^31^, we hypothesize that *Cannabis* growers and breeders have been assigning labels to cultivars primarily on the basis of aroma profiles and purported effects, rather than genetic ancestry or overall chemical similarity. The primary differences between cultivars labelled as Sativa and Indica may thus be driven by a small set of genomic regions controlling the concentrations of a small number of contrasting aromas. To examine this, we conducted a genome-wide association study (GWAS) of the 40 chemicals examined here (Supplementary Fig. 2 and Supplementary Table 2).

**Extended Data Fig. 4:**
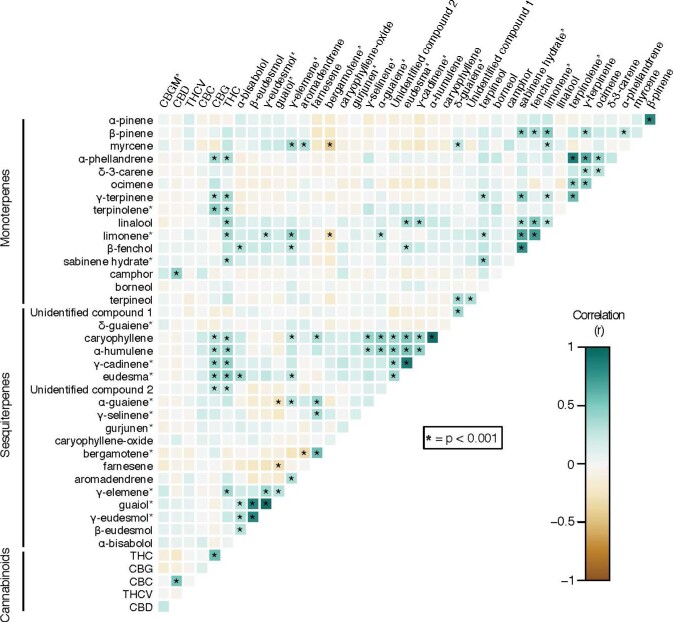
Chemical correlation heatmap. Heatmap displaying the Pearson correlation between the concentrations of the 40 terpenes and cannabinoids.

We identified three regions of the Cannabis genome associated with the four terpenes most strongly associated with Sativa–Indica labelling (Fig. 2). The optimal model from the multilocus mixed linear model (MLMM) GWAS for myrcene identified two significantly associated SNPs 1.2 megabases apart that tag independent blocks of linkage disequilibrium (LD) on the proximal end of chromosome 5 (Fig. 2b). The first SNP (chr5:1348048) is located 6.4 kilobases (kb) from a block of terpene synthase genes composed of four copies of *TPS30*, which is known to encode myrcene synthase^12^ (Supplementary Table 3). The second SNP (chr5:2576403) is 46.7 kb from another tandem array of terpene synthase genes spanning ~200 kb (Supplementary Table 3). Within this gene cluster are two sequences highly similar to the myrcene synthase gene, *TPS3* (refs. ^12,13^). These observations suggest that myrcene synthesis is mediated by genetic variants at two independent terpene synthase gene clusters on chromosome 5. The other three sesquiterpenes (guaiol, β-eudesmol and γ-eudesmol) strongly associated with Sativa–Indica labelling are correlated with each other (Extended Data Fig. 4) and share a common GWAS hit on chromosome 6: the single SNP identified from the MLMM (chr6:76790611) is 51.9 kb from a gene cluster comprising sesquiterpene synthase genes related to TPS7FN (δ-selinene synthase), TPS8FN (γ-eudesmol/valencene synthase)^12^ and TPS20CT^13^ (hedycaryol synthase) (Fig. 2c and Supplementary Table 3).

Our results demonstrate that the Sativa–Indica scale currently used to label *Cannabis* poorly captures overall genomic and metabolomic variation. *Cannabis* labelling is instead probably driven primarily by a small number of key terpenes whose concentrations contribute to the characteristic aromas commonly associated with Sativa and Indica and whose variation we genetically mapped to tandem arrays of terpene synthase genes on chromosomes 5 and 6. While the vernacular labels ‘Sativa’ and ‘Indica’ are derived from taxonomic names that were originally used to categorize plants according to ancestry^4^, these terms have been co-opted by contemporary *Cannabis* culture and now probably reflect locus-specific genetic variation affecting terpene synthesis. Our results suggest that a practical and reliable classification system for *Cannabis* that is consistent with contemporary understanding of the terms ‘Sativa’ and ‘Indica’ may be achievable by quantifying a small number of terpenes and/or genotyping genetic markers associated with key *Cannabis* aromas.

## Methods

### Samples

The samples come from a previous study of 460 *Cannabis* chemotypes^16^. The samples were collected from Bedrocan International BV (*n* = 37), HempFlax (*n* = 205) and Dutch ‘coffee shops’ either directly or indirectly through the TRIMBOS Institute (*n* = 55). Samples labelled as ‘Hemp’ were excluded from the analysis. We retained and analysed 297 samples that were classified along a five-point scale according to ancestries reported by the sources: ‘Sativa’ (100% Sativa), ‘Hybrid-Sativa’ (75% Sativa, 25% Indica), ‘Hybrid’ (50% Sativa, 50% Indica), ‘Hybrid-Indica’ (25% Sativa, 75% Indica) and ‘Indica’ (100% Indica). These five groups were encoded as 1 (100% Sativa) to 5 (100% Indica) for the statistical analyses described below.

### Gas chromatography

A total of 297 samples were previously quantified for terpene and cannabinoid content, and we conduct a re-analysis of these data here. The chemical analyses of the samples are described in detail in ref. ^16^. Briefly, for each sample, 500 mg of ground homogenized dried flower material was mixed with 40 ml of ethanol, agitated for 10 minutes and centrifuged. The supernatant was collected, and the process was repeated twice more on the pellet. An internal standard consisting of 200 μl of 1% solution of 1-octanol was added to the combined supernatant, the volume was adjusted to 100 ml with ethanol and the combined sample was centrifuged again. The combined sample was analysed using an Agilent GC 6890 series (Agilent Technologies) equipped with a 7683 autosampler and a flame ionizing detector. The instrument was equipped with a DB-5 column (length, 30 m; internal diameter, 0.25 mm; film thickness, 0.25 μm; J&W Scientific). Peaks from the sample chromatograms were manually integrated, and the peak area was recorded with correction for the internal standard peak area. Peak identification was conducted by analysing selected samples using GC–MS and then comparing compounds’ mass spectra and retention times with authentic standards and literature reports as described in ref. ^16^. Compounds without authentic standards are marked with an asterisk in the figures to indicate that they were tentative identifications. Peak areas of monoterpenes, sesquiterpenes and cannabinoids were quantified (in mg per g of plant material) using calibrated standards of β-pinene, α-humulene and CBD, respectively. We re-assessed the compound identifications in Hazekamp et al.^16^, and in certain cases we renamed compounds on the basis of the inability to distinguish stereoisomers using a DB-5 column. For example, in the case of the compound listed by Hazekamp et al.^16^ as ‘(−)-linalool’, we renamed this to ‘linalool’. There are also two compounds that could not be reliably identified; they are listed as ‘unidentified compounds’ (Supplementary Table 3). THC, δ-8-THC and CBN were combined into a single value, ‘Total THC’, because δ-8-THC and CBN are degradation products of THC. Peaks of *R*-limonene and β-phellandrene were indistinguishable and were therefore combined into a single value and reported as ‘limonene’. Thymoquinone, geraniol, thymol and carvacrol were removed because they were not present in any samples, and cineol was removed because it was present in only one sample. Pearson correlations were calculated between each pair of chemicals using the cor.test function in R v.3.5.1^32^. According to previous work^33^, the samples analysed here were nearly all drug-type *Cannabis* (that is, type I) (Extended Data Fig. 1), except nine samples with THC > 0.3% and CBD > 0.5% (that is, type II).

### Genomic analysis

Whole-genome DNA was extracted using a NucleoSpin 96 Plant II kit (Machery-Nagel) and quantified using the QuantiFluor dsDNA System and the GloMax-Multi + Microplate Multimode Reader with Instinct (Promega). Genotyping-by-sequencing libraries were prepared using the restriction enzyme ApeKI^34^, and the libraries were sequenced on two lanes of an Illumina Hi-Seq 4000 (Illumina). The DNA sequence data are available as NCBI BioProject PRJNA713792. Calling of SNPs was performed in TASSEL (v.5.0)^35^ by aligning to the CBDRx reference genome^8^. SNP calling was performed before the implementation of the new chromosome numbering of the CBDRx genome in April 2020. Chromosomes were recoded for analyses to reflect the new chromosome numbering system. We used VCFtools (v.0.1.15)^36^ to retain only bi-allelic SNPs and samples with <70% missing data, which resulted in 155 remaining samples and 284,988 SNPs. Genotype imputation was performed using LinkImputeR^37^ with a minor allele frequency threshold of 0.01, a minimum read depth for masking of 20 and the number of masked genotypes set to 5,000. We chose to impute with a minimum read count of 2 and a maximum missingness threshold of 70%, which resulted in an imputation accuracy of 92.88%. After imputation, 149 samples remained. An additional 12 samples were removed because they had no phenotype data. This resulted in a final set of 137 samples with both genetic and chemical data. The SNP data were filtered using PLINK (v.1.90)^38^ to exclude SNPs with a minor allele frequency less than 0.05 and SNPs with excess heterozygosity resulting in Hardy–Weinberg *P* values less than 1 × 10^−5^. The final SNP dataset used for GWAS consisted of 116,296 SNPs from 137 samples. For PCA, 1,257 unanchored SNPs were removed, and the remaining 115,039 SNPs were LD-pruned using PLINK (command: –indep-pairwise 10 3 0.5), resulting in 80,939 SNPs.

### Genetic and chemical analysis

The chemical distance between cultivars was calculated as the Euclidean distance using the ‘dist’ function in R from the matrix of metabolomic data—that is, 40 terpenes and cannabinoids quantified across 297 samples. The genetic similarity between samples was calculated as an inverse identity-by-state matrix generated in PLINK. The correlations between the matrices were computed using a Mantel test in R^32^ by first reducing the chemical matrix to the 137 samples with both chemical and genetic datasets. PCA was performed on the scaled genetic and chemical data using the prcomp function in R. To calculate the variance in labelling explained by the chemical and genetic data, linear models including the top ten PCs from the genetic data, the chemical data and both the chemical and genetic datasets together were performed. Pearson correlations between chemical concentration and the 1-to-5 Sativa–Indica scale were performed with the cor.test function in R. A Bonferroni correction was applied to the *P* values from the correlation test between chemical concentration and the Sativa–Indica scale.

### Genome-wide association

We performed GWAS for 40 terpene and cannabinoid phenotypes, using both normalized and non-normalized data. Normalizing was conducted to generate values for a chemical concentration in a sample relative to the total abundance of its chemical class (that is, monoterpene, sesquiterpene or cannabinoid) in that sample. Thus, a sample’s myrcene content was divided by the total concentration of all monoterpenes in that sample to generate a normalized value for myrcene. GWAS was performed using an MMLM^39^ accounting for relatedness using a kinship matrix created in TASSEL (v.5.0)^35^. The MLMM incorporates significant SNPs as cofactors using stepwise regression (maxsteps = 10), and the optimal model was chosen on the basis of the extended Bayesian information criterion. We also present the first step of the MLMM, which is equivalent to an MLM where relatedness is accounted for but no SNPs are included as cofactors. Using the simpleM^40^ package in R, the effective number of independent tests (*M*_eff_) was generated, and the threshold for significance was then calculated using −log_10_(*α*/*M*_eff_), where *α* = 0.05. Quantile–quantile and Manhattan plots were created using the qq function in R. Genomic regions with significant GWAS hits were explored, and the physical locations of genes within these regions were retrieved using annotations from the CBDRx reference genome^8^ in Geneious Prime (v.2020.1.2). The GWAS results and LD regions of interest were visualized using code adapted from ref. ^41^.

### Reporting Summary

Further information on research design is available in the Nature Research Reporting Summary linked to this article.

## Supplementary information

Supplementary Information Supplementary Figs. 1 and 2.

Reporting Summary

Supplementary Tables Supplementary Table 1: Chemical concentrations and labels across 297 *Cannabis* samples. The asterisks denote chemicals with tentative identifications. Supplementary Table 2: Significant SNPs from the MLMM GWAS for myrcene and three sesquiterpenes. Only SNPs identified as significantly (*P* < 6.69 × 10^−7^) associated with a trait according to the MLMM GWAS are shown. The genomic coordinates and annotations, *P* value, *R*^2^ value and nearby candidate genes are shown. Supplementary Table 3: A list of compound names identified by Hazekamp et al.^16^, a list of authentic standards used for compound identification and a list of compound names based on re-analysis of the methods used in Hazekamp et al.^16^.

## Data Availability

The authors declare that the data supporting the findings are available within the paper. The sequence data are available in the NCBI Short Read Archive under BioProject No. PRJNA713792. The genotype files are available at 10.5061/dryad.gqnk98smm.

## Code availability

All code used for the analyses is available through GitHub at https://github.com/MylesLab/cannabis-labelling.
