# Supplementary material for: Cannabis labelling is associated with genetic variation in terpene synthase genes
Source: Nat Plants. 2021 Oct 14;7(10):1330–4. doi: 10.1038/s41477-021-01003-y (PMC8516649; doi:10.1038/s41477-021-01003-y)
Supplement: Supplementary file 1 — Supplementary Figs. 1 and 2. [file 41477_2021_1003_MOESM1_ESM.pdf]

nature portfolio

https://doi.org/10.1038/s41477-021-01003-y

Supplementary information

Cannabis labelling is associated with genetic variation in terpene synthase genes

In the format provided by the authors and unedited

Supplemental Figure 1: Boxplots of chemical concentrations across the Sativa-Indica scale, n= 68 ‘Sativa’, n= 27 ‘Sativa-Hybrid’, n= 115 ‘Hybrid’, n= 25 ‘Indica-Hybrid’ and n= 62 ‘Indica’ samples. The lower and upper bounds of the boxplots correspond to the 25% and 75% quantiles, the minima and maxima of whiskers extend to the lowest and largest value, respectively, within 1.5 times the inter-quartile range, and the centre of the boxes represent the median value. Outliers beyond the whiskers are plotted individually. Y-axis is expressed as milligrams per gram. Asterisks denote chemicals with tentative identifications.

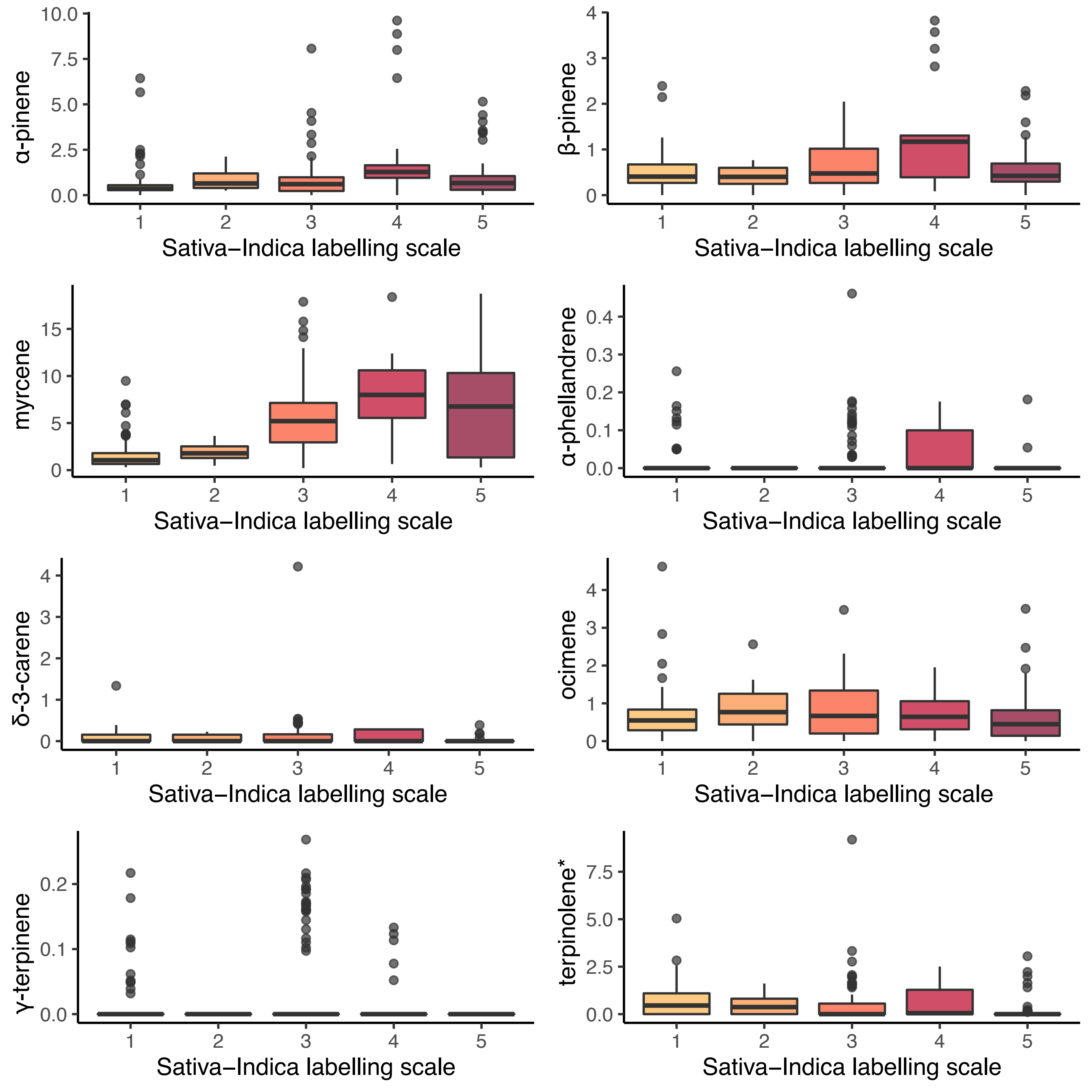
α-pinene
β-pinene
myrcene
α-phellandrene
δ-3-carene
ocimene
γ-terpinene
terpinolene*
Sativa−Indica labelling scale
1
2
3
4
5

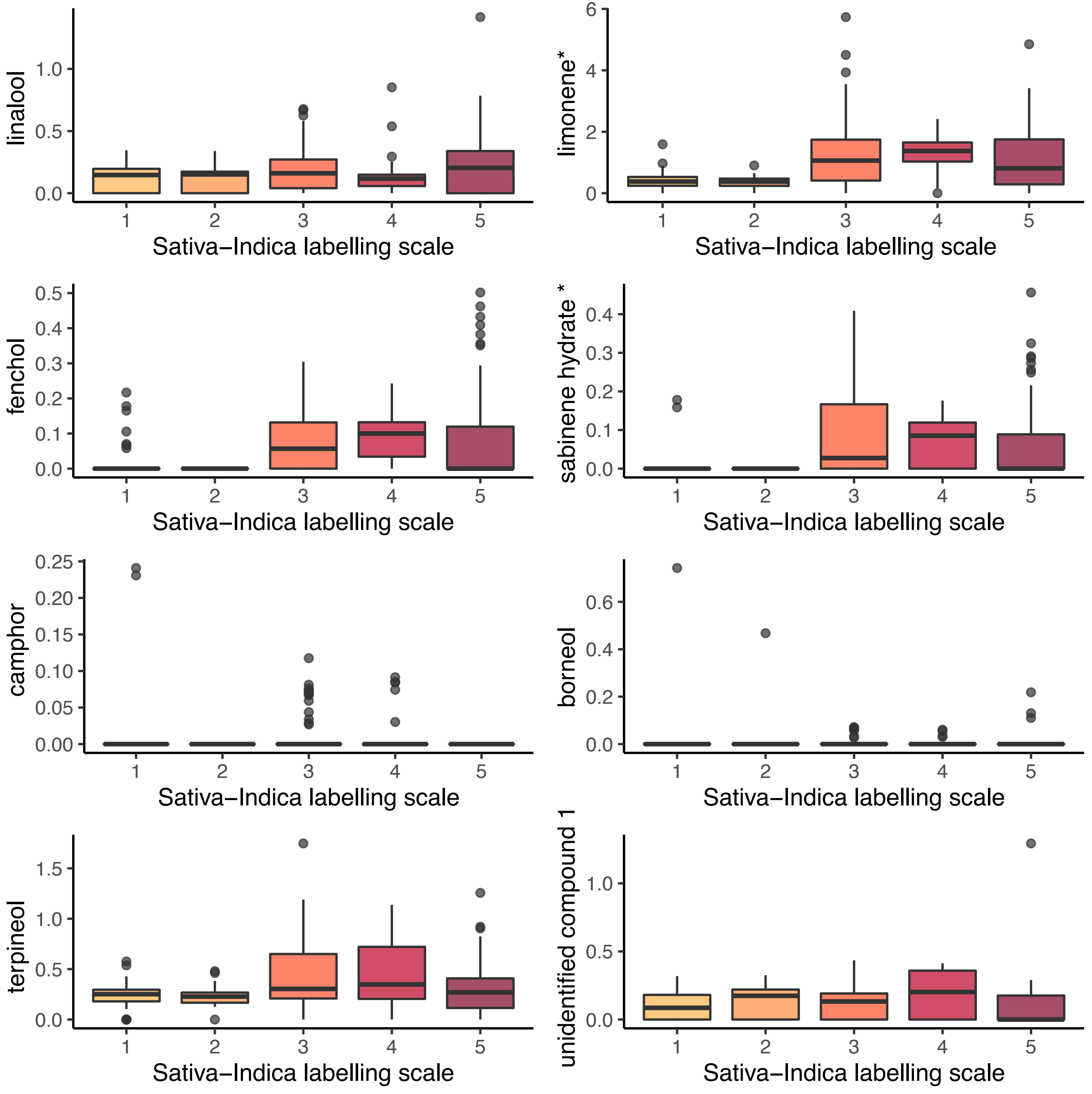
linalool
limonene*
fenchol
sabinene hydrate*
camphor
borneol
terpineol
unidentified compound 1
Sativa–Indica labelling scale
1
2
3
4
5

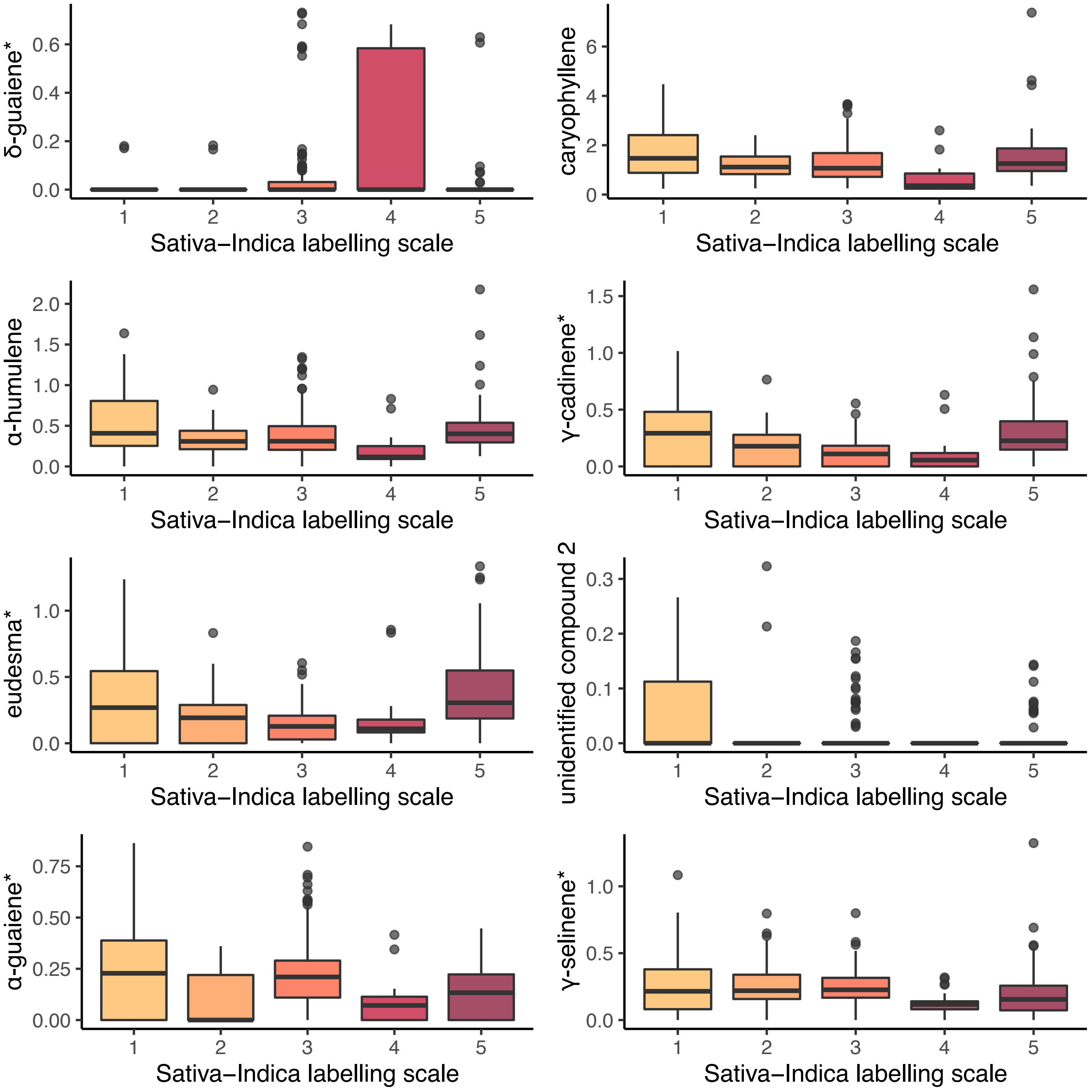
δ-guaiene*
caryophyllene
α-humulene
γ-cadinene*
eudesma*
unidentified compound 2
α-guaiene*
γ-selinene*
Sativa–Indica labelling scale

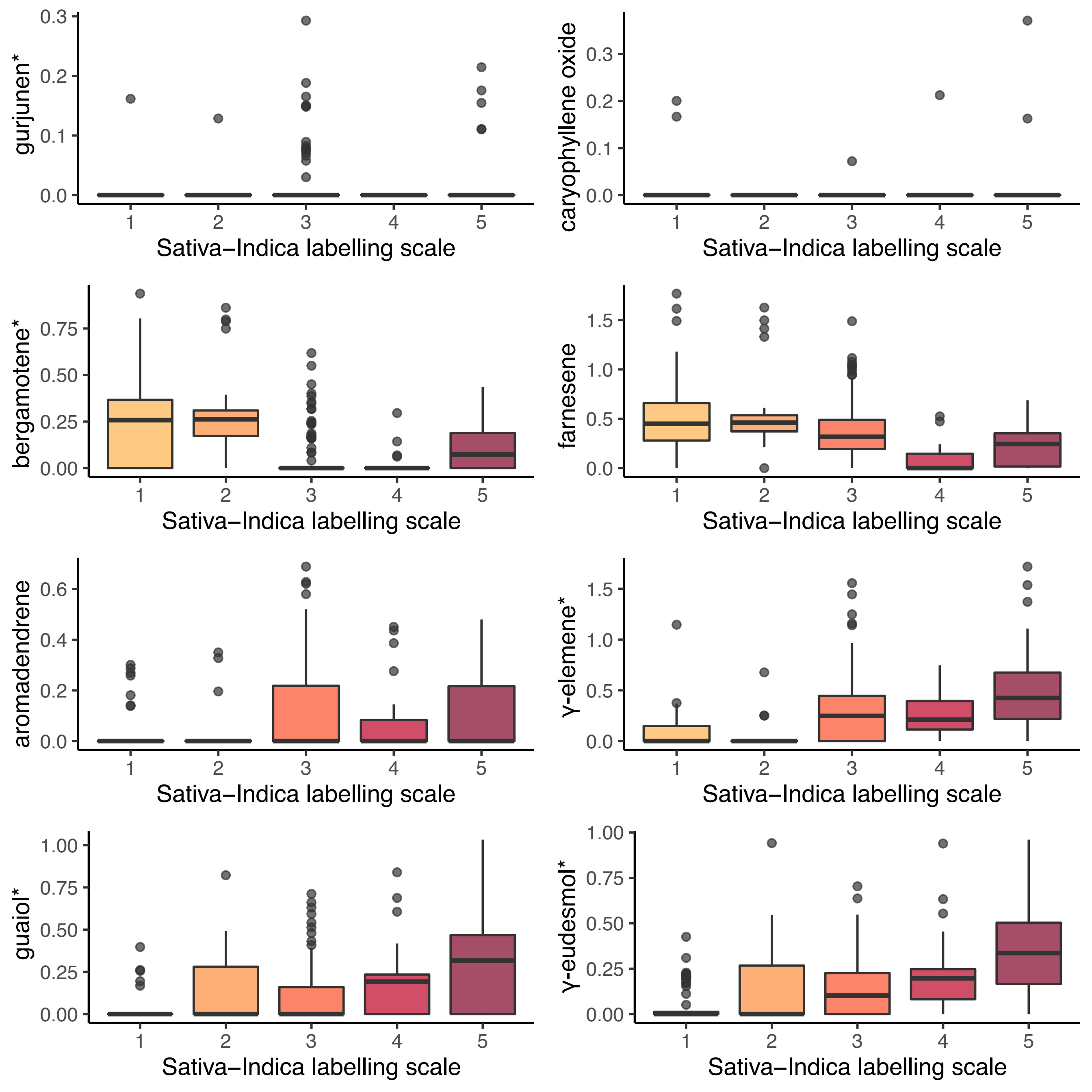

gurjunen*
0.0
0.1
0.2
0.3
1
2
3
4
5
Sativa−Indica labelling scale
caryophyllene oxide
0.0
0.1
0.2
0.3
bergamotene*
0.00
0.25
0.50
0.75
farnesene
0.0
0.5
1.0
1.5
aromadendrene
0.0
0.2
0.4
0.6
γ-elemene*
guaiol*
1.00
γ-eudesmol*

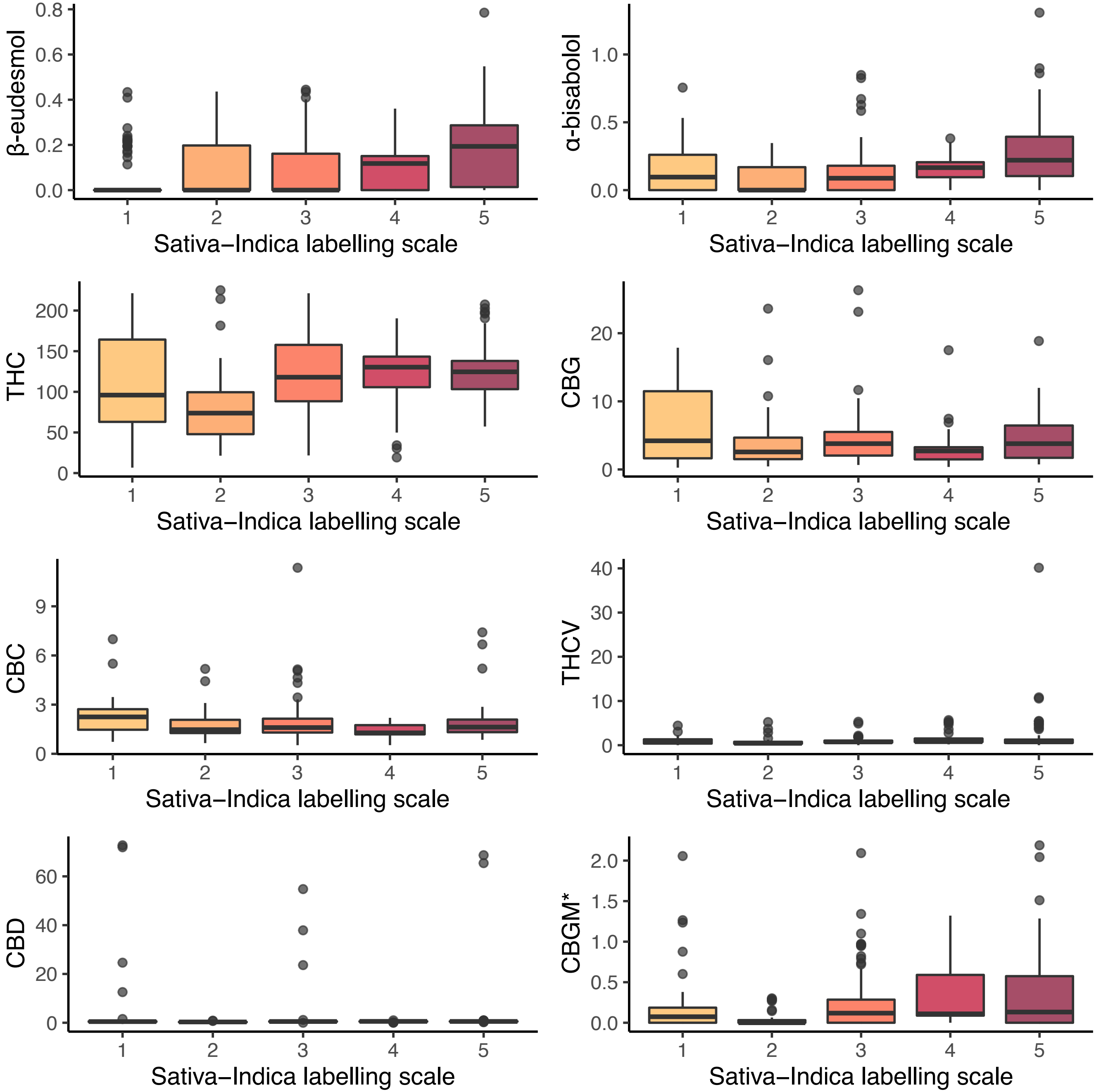

β-eudesmol
α-bisabolol
THC
CBG
CBC
THCV
CBD
CBGM*
Sativa–Indica labelling scale
1 2 3 4 5

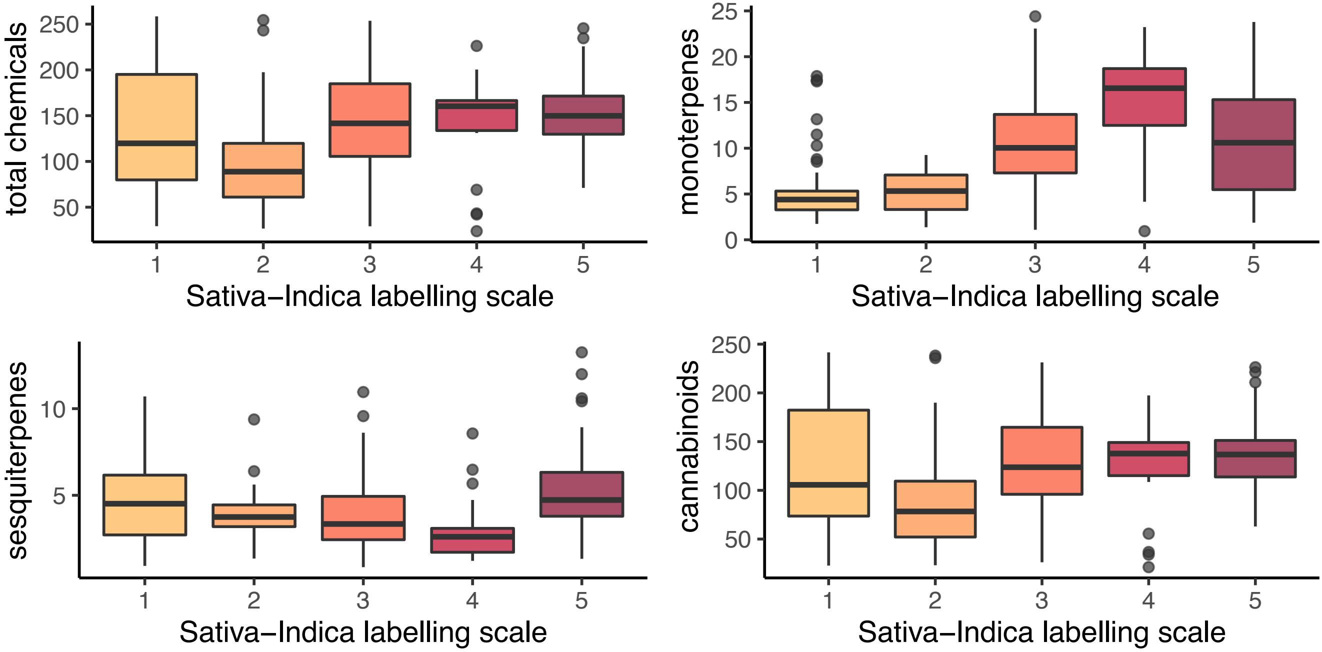

total chemicals
250
200
150
100
50
1
2
3
4
5
Sativa−Indica labelling scale
monoterpenes
25
20
15
10
5
0
1
2
3
4
5
Sativa−Indica labelling scale
sesquiterpenes
10
5
1
2
3
4
5
Sativa−Indica labelling scale
cannabinoids
250
200
150
100
50
1
2
3
4
5
Sativa−Indica labelling scale

Supplemental Figure 2: Manhattan plots from the standard mixed linear model (MLM) and the multi-locus mixed-linear model (MLMM) GWAS and QQ plots from the standard MLM GWAS. Asterisks denote chemicals with tentative identifications.

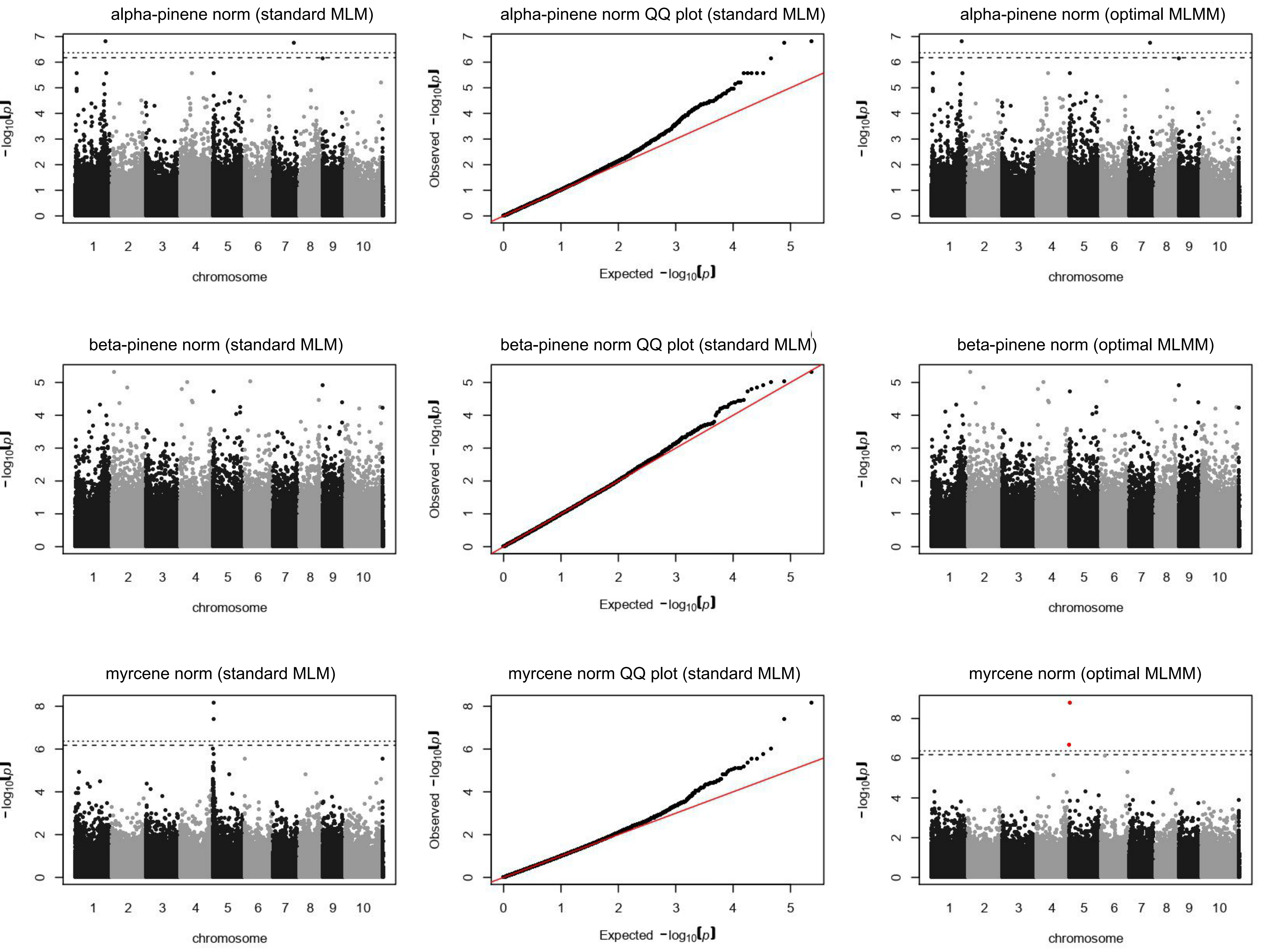

alpha-pinene norm (standard MLM)
alpha-pinene norm QQ plot (standard MLM)
alpha-pinene norm (optimal MLMM)
beta-pinene norm (standard MLM)
beta-pinene norm QQ plot (standard MLM)
beta-pinene norm (optimal MLMM)
myrcene norm (standard MLM)
myrcene norm QQ plot (standard MLM)
myrcene norm (optimal MLMM)
$-\log_{10}(p)$
Observed $-\log_{10}(p)$
Expected $-\log_{10}(p)$
chromosome
1 2 3 4 5 6 7 8 9 10

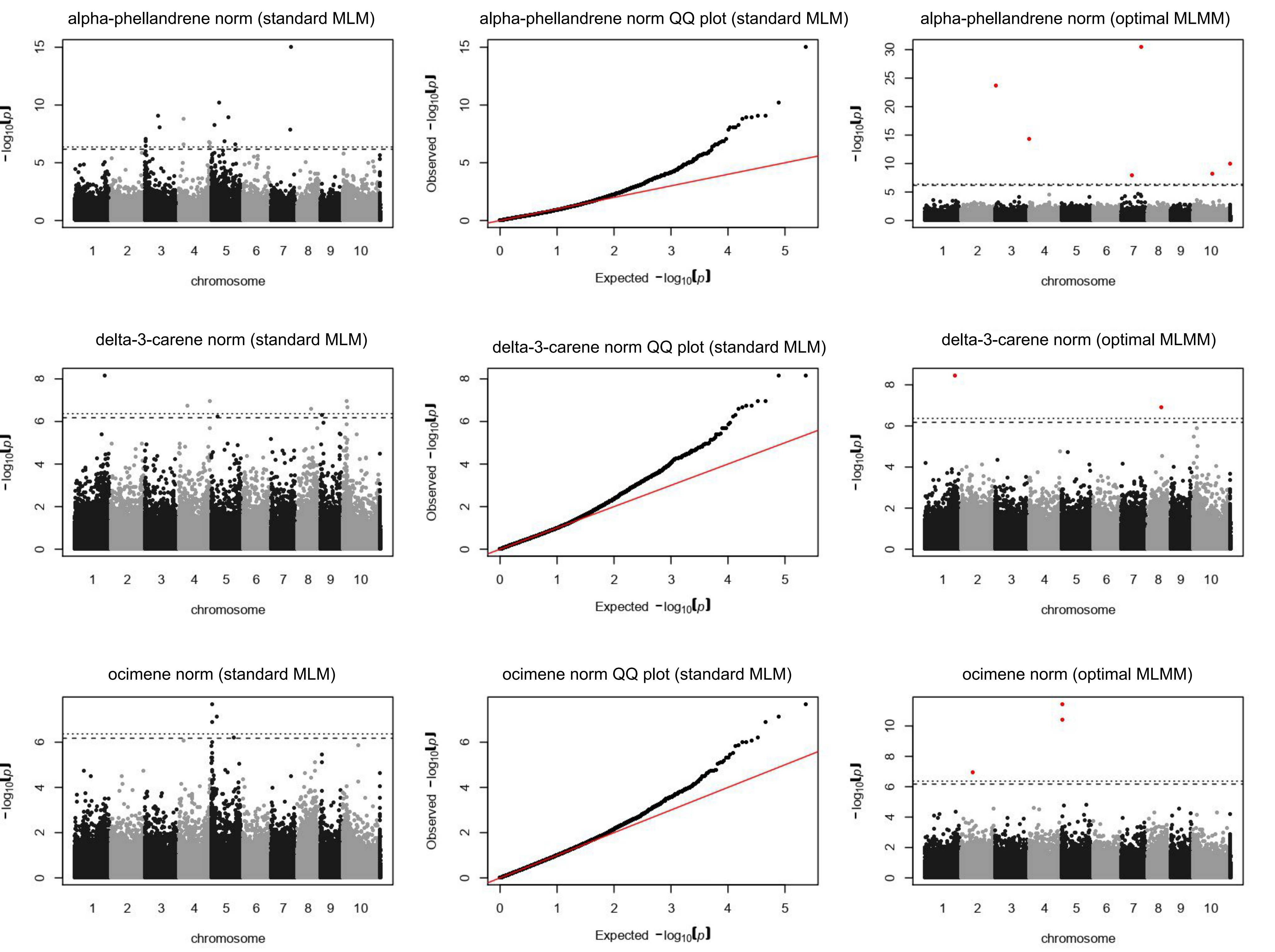

alpha-phellandrene norm (standard MLM)
alpha-phellandrene norm QQ plot (standard MLM)
alpha-phellandrene norm (optimal MLMM)
delta-3-carene norm (standard MLM)
delta-3-carene norm QQ plot (standard MLM)
delta-3-carene norm (optimal MLMM)
ocimene norm (standard MLM)
ocimene norm QQ plot (standard MLM)
ocimene norm (optimal MLMM)
chromosome
Expected −log10(p)
Observed −log10(p)
−log10(p)

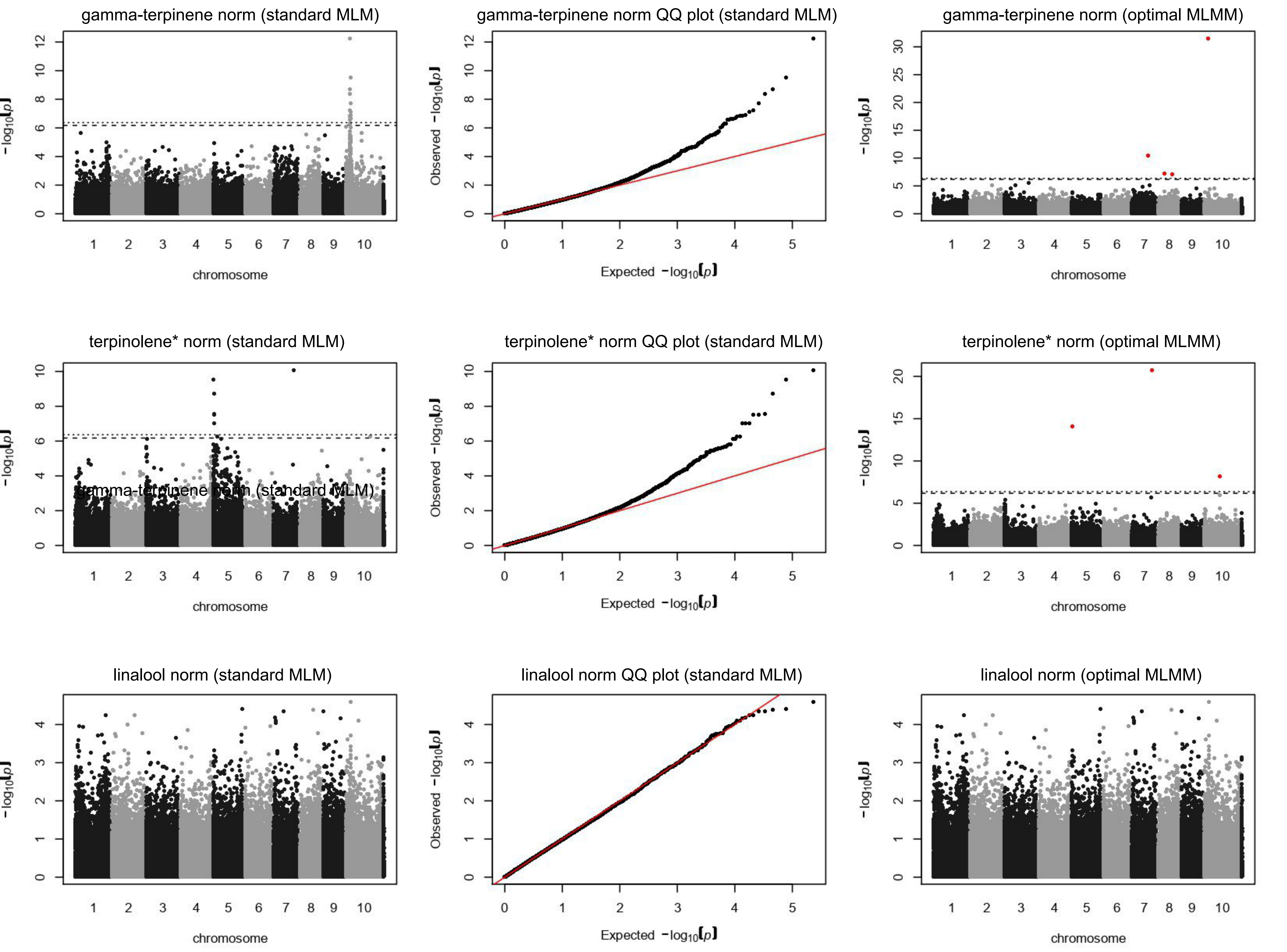
gamma-terpinene norm (standard MLM)
gamma-terpinene norm QQ plot (standard MLM)
gamma-terpinene norm (optimal MLMM)
terpinolene* norm (standard MLM)
gamma-terpinene norm (standard MLM)
terpinolene* norm QQ plot (standard MLM)
terpinolene* norm (optimal MLMM)
linalool norm (standard MLM)
linalool norm QQ plot (standard MLM)
linalool norm (optimal MLMM)
$-\log_{10}(p)$
Observed $-\log_{10}(p)$
Expected $-\log_{10}(p)$
chromosome

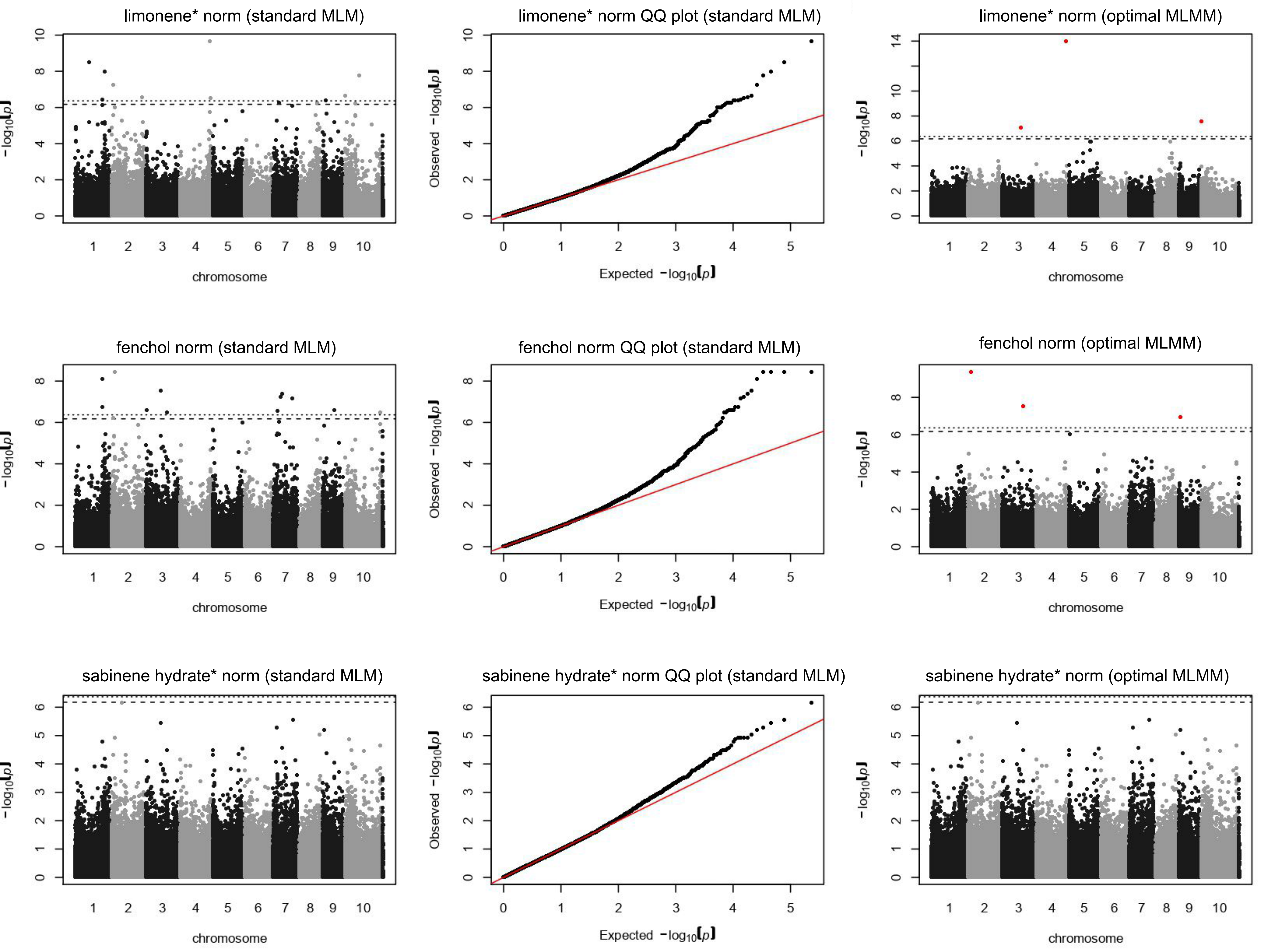

limonene* norm (standard MLM)
limonene* norm QQ plot (standard MLM)
limonene* norm (optimal MLMM)
fenchol norm (standard MLM)
fenchol norm QQ plot (standard MLM)
fenchol norm (optimal MLMM)
sabinene hydrate* norm (standard MLM)
sabinene hydrate* norm QQ plot (standard MLM)
sabinene hydrate* norm (optimal MLMM)
chromosome
Expected −log10(p)
Observed −log10(p)
−log10(p)

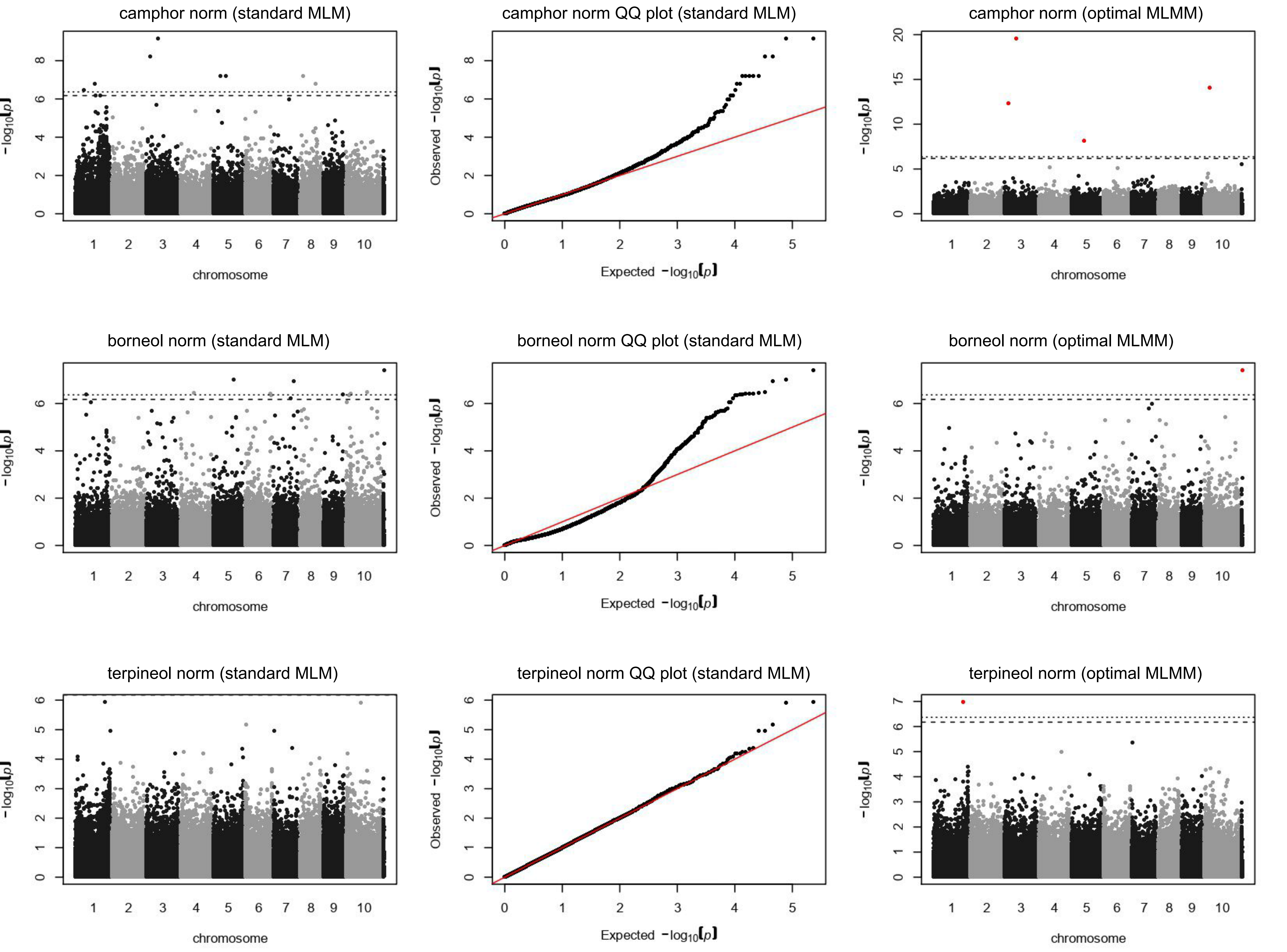

camphor norm (standard MLM)
camphor norm QQ plot (standard MLM)
camphor norm (optimal MLMM)
borneol norm (standard MLM)
borneol norm QQ plot (standard MLM)
borneol norm (optimal MLMM)
terpineol norm (standard MLM)
terpineol norm QQ plot (standard MLM)
terpineol norm (optimal MLMM)
chromosome
Expected −log10(p)
Observed −log10(p)
−log10(p)

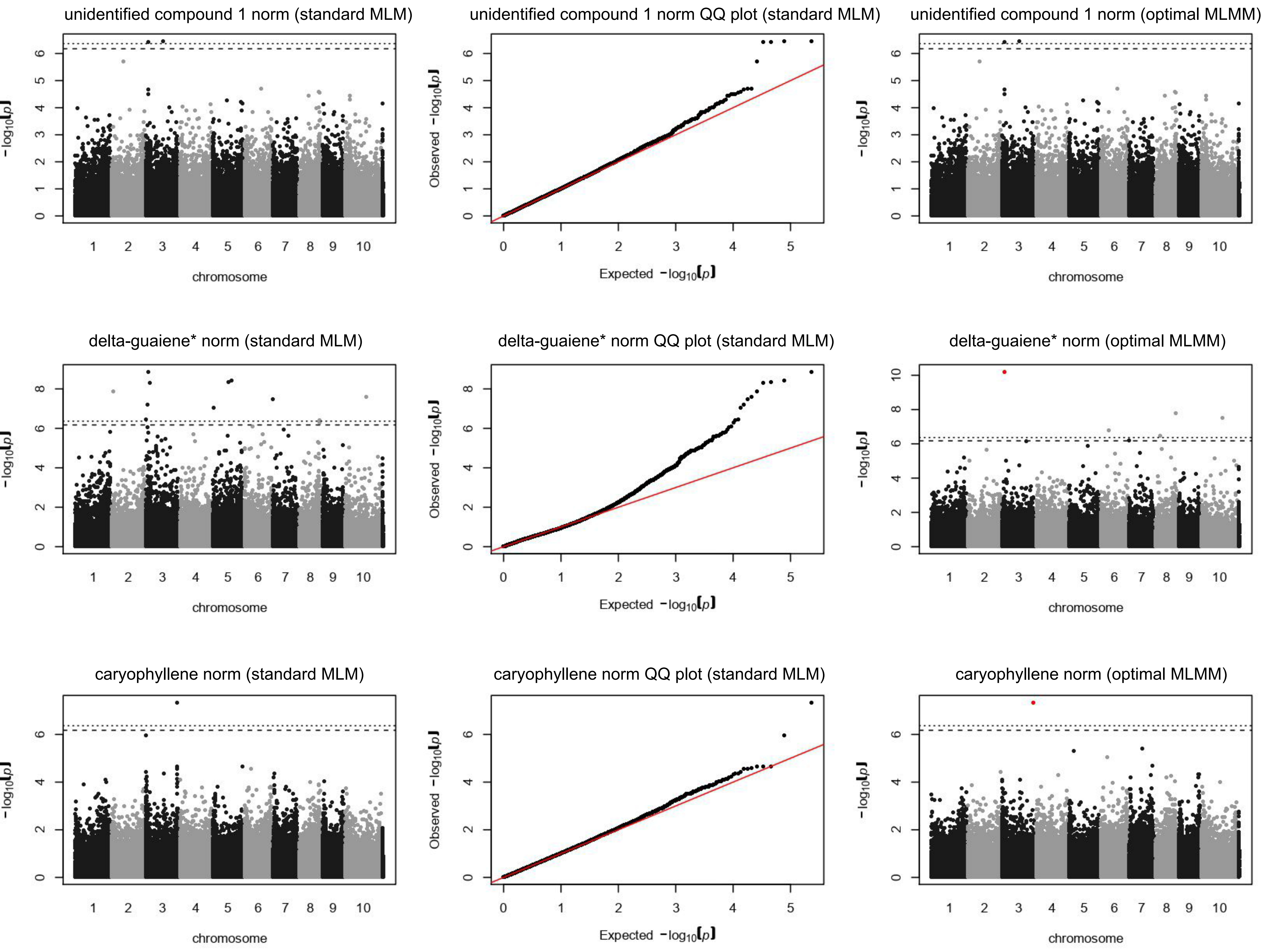
unidentified compound 1 norm (standard MLM)
unidentified compound 1 norm QQ plot (standard MLM)
unidentified compound 1 norm (optimal MLMM)
delta-guaiene* norm (standard MLM)
delta-guaiene* norm QQ plot (standard MLM)
delta-guaiene* norm (optimal MLMM)
caryophyllene norm (standard MLM)
caryophyllene norm QQ plot (standard MLM)
caryophyllene norm (optimal MLMM)
$-\log_{10}(p)$
Observed $-\log_{10}(p)$
Expected $-\log_{10}(p)$
chromosome

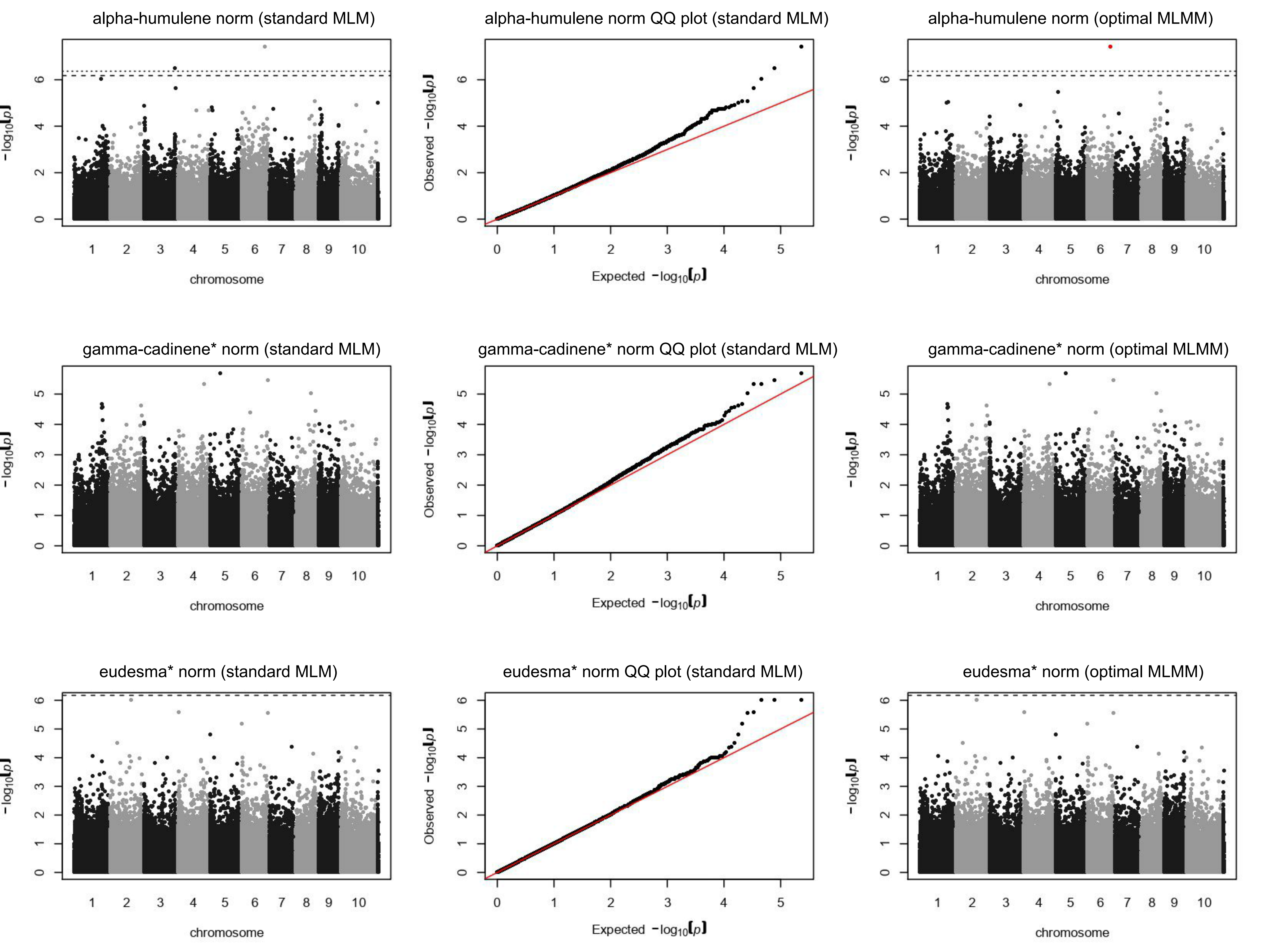
alpha-humulene norm (standard MLM)
alpha-humulene norm QQ plot (standard MLM)
alpha-humulene norm (optimal MLMM)
gamma-cadinene* norm (standard MLM)
gamma-cadinene* norm QQ plot (standard MLM)
gamma-cadinene* norm (optimal MLMM)
eudesma* norm (standard MLM)
eudesma* norm QQ plot (standard MLM)
eudesma* norm (optimal MLMM)
$-\log_{10}(p)$
chromosome
Observed $-\log_{10}(p)$
Expected $-\log_{10}(p)$

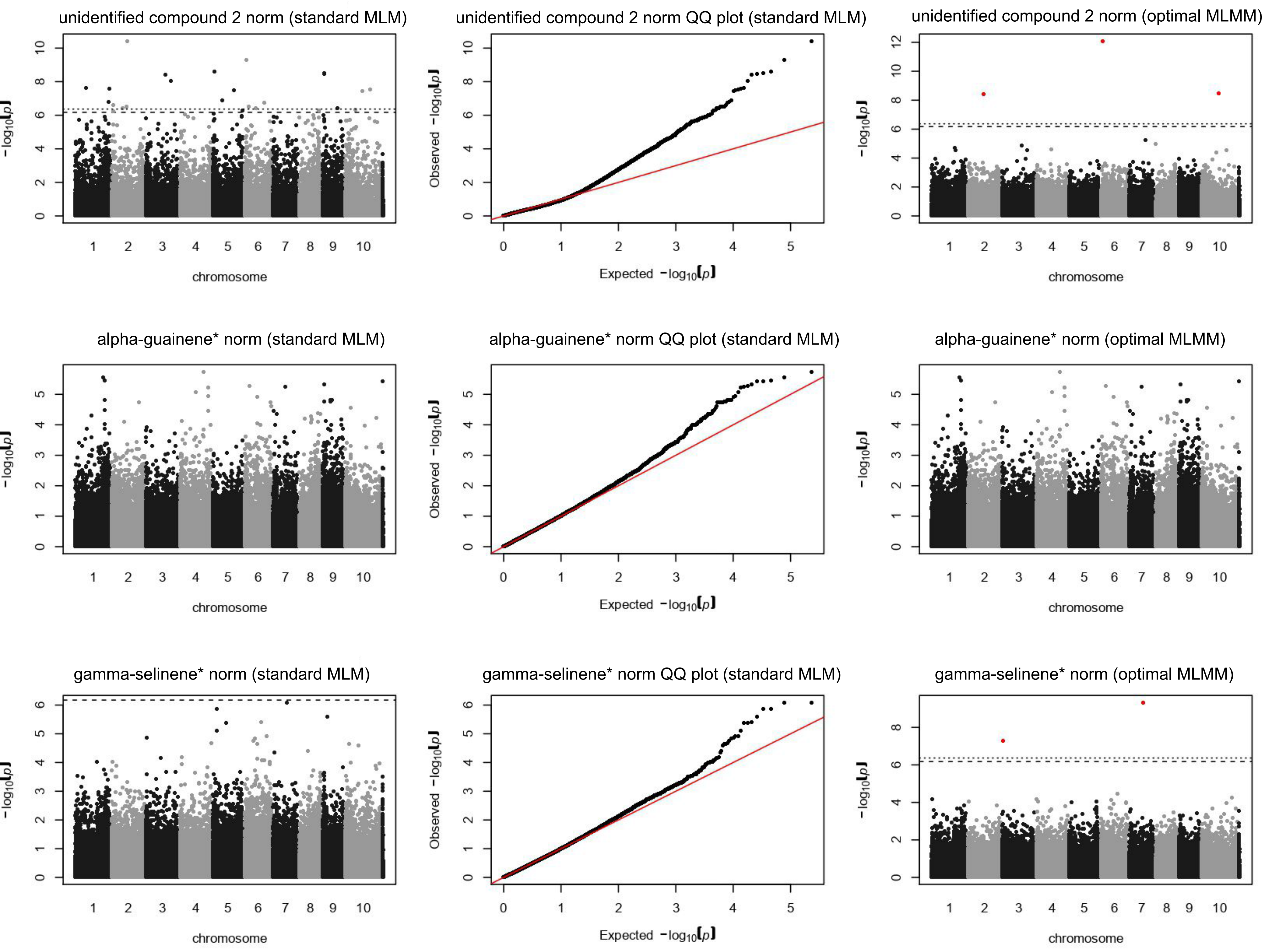
unidentified compound 2 norm (standard MLM)
unidentified compound 2 norm QQ plot (standard MLM)
unidentified compound 2 norm (optimal MLMM)
alpha-guainene* norm (standard MLM)
alpha-guainene* norm QQ plot (standard MLM)
alpha-guainene* norm (optimal MLMM)
gamma-selinene* norm (standard MLM)
gamma-selinene* norm QQ plot (standard MLM)
gamma-selinene* norm (optimal MLMM)
$-\log_{10}(p)$
Observed $-\log_{10}(p)$
Expected $-\log_{10}(p)$
chromosome

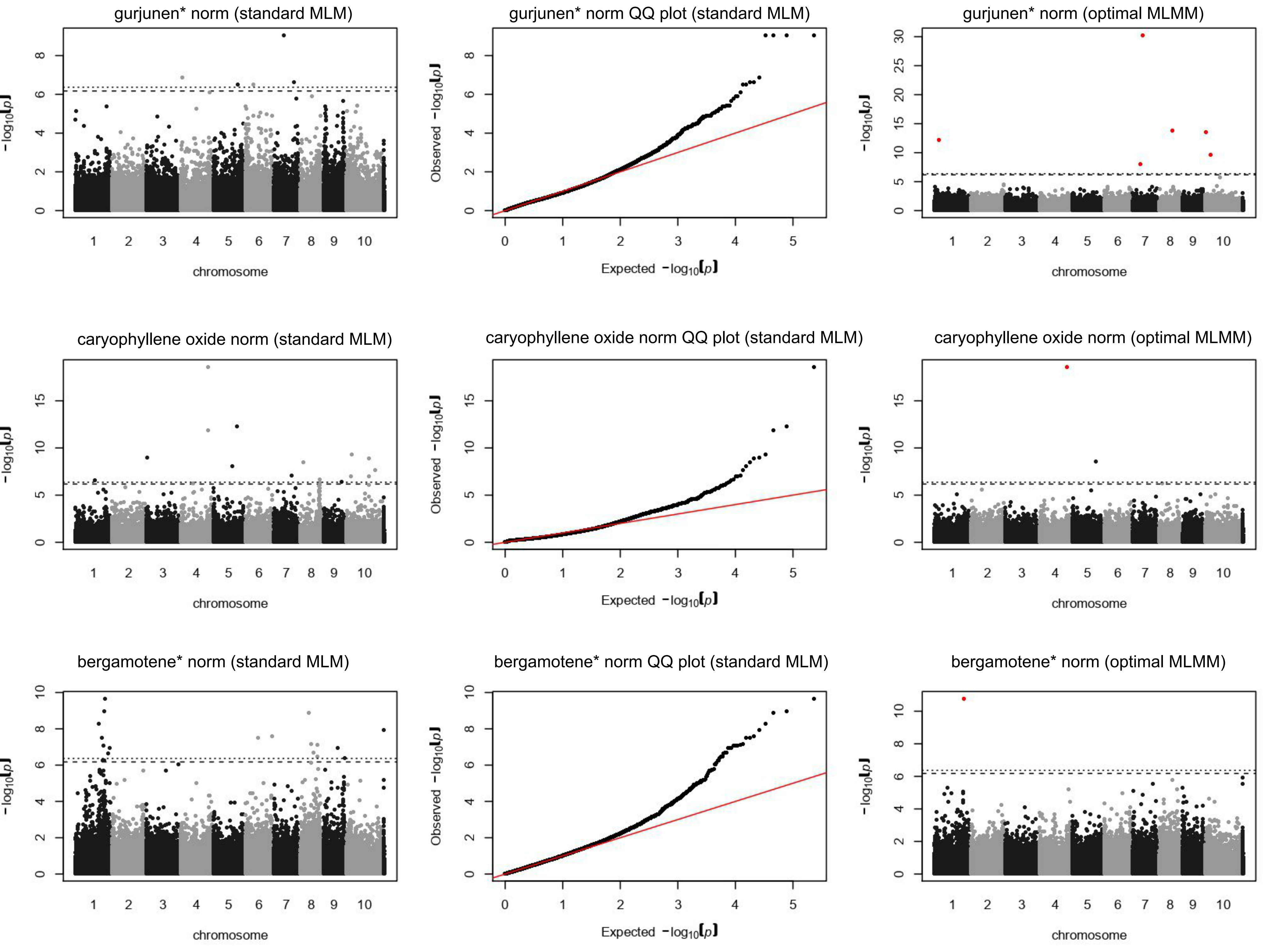

gurjunen* norm (standard MLM)
gurjunen* norm QQ plot (standard MLM)
gurjunen* norm (optimal MLMM)
caryophyllene oxide norm (standard MLM)
caryophyllene oxide norm QQ plot (standard MLM)
caryophyllene oxide norm (optimal MLMM)
bergamotene* norm (standard MLM)
bergamotene* norm QQ plot (standard MLM)
bergamotene* norm (optimal MLMM)
−log10(p)
Observed −log10(p)
Expected −log10(p)
chromosome

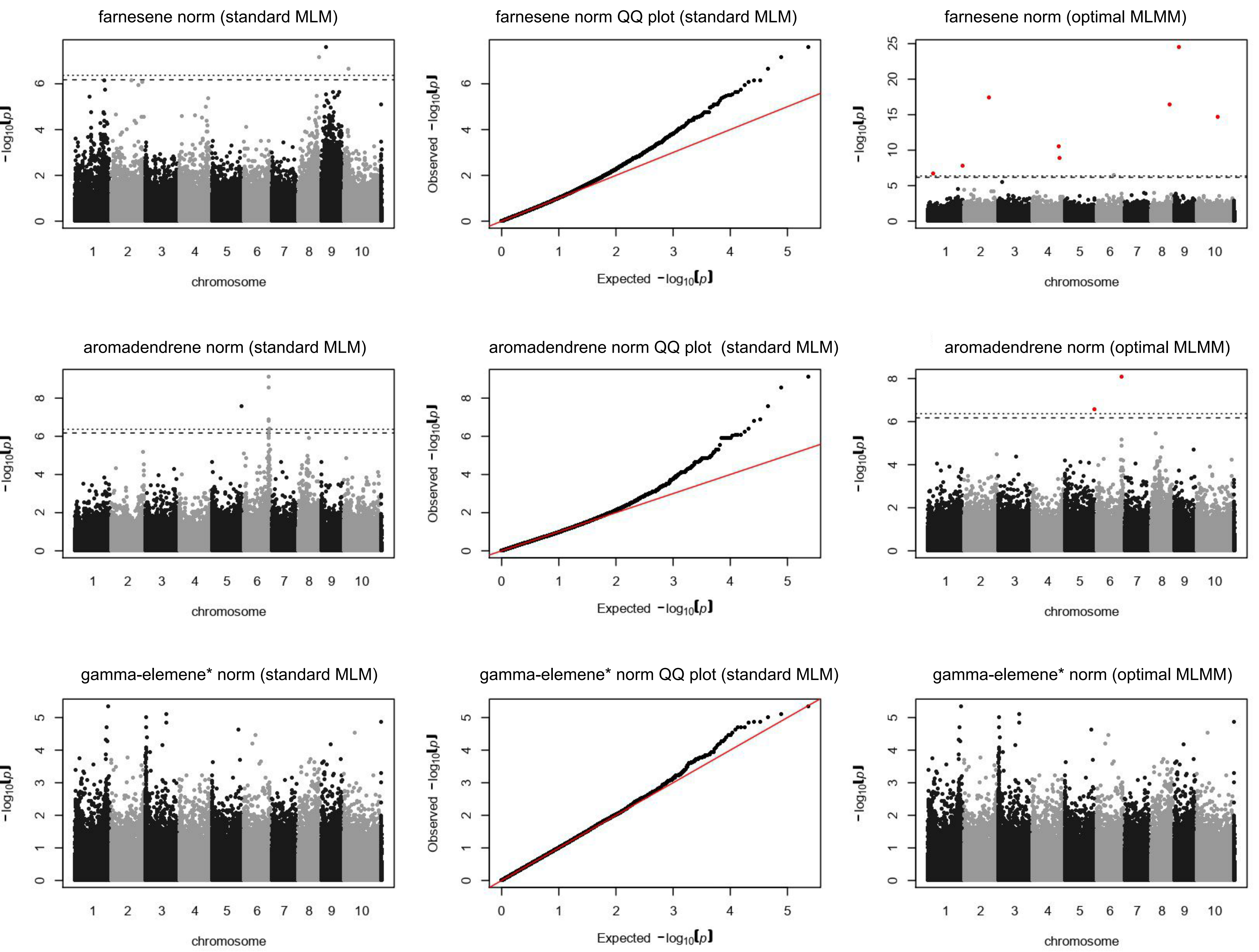

farnesene norm (standard MLM)
farnesene norm QQ plot (standard MLM)
farnesene norm (optimal MLMM)
aromadendrene norm (standard MLM)
aromadendrene norm QQ plot (standard MLM)
aromadendrene norm (optimal MLMM)
gamma-elemene* norm (standard MLM)
gamma-elemene* norm QQ plot (standard MLM)
gamma-elemene* norm (optimal MLMM)
$-\log_{10}(p)$
Observed $-\log_{10}(p)$
Expected $-\log_{10}(p)$
chromosome

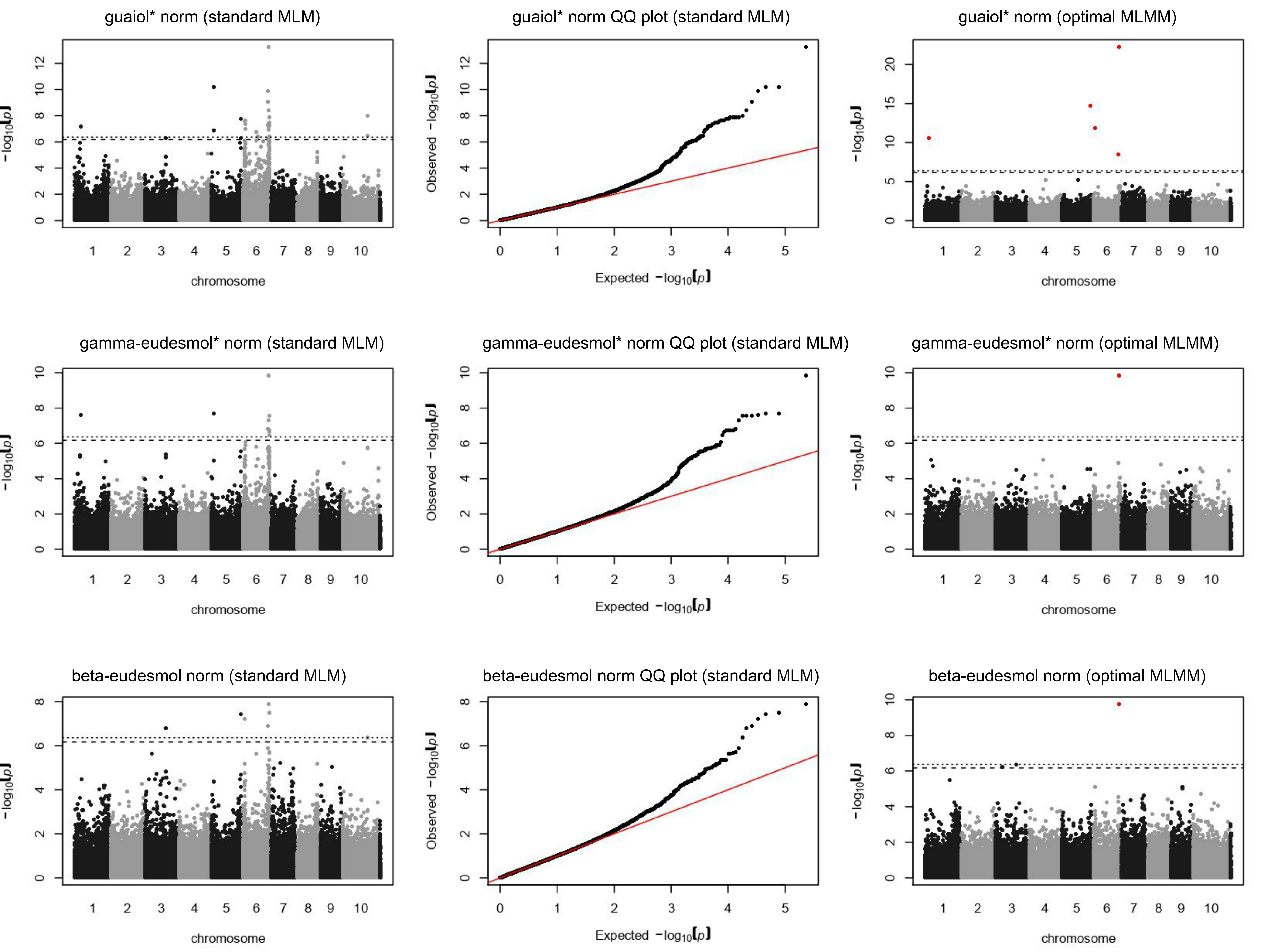
guaiol* norm (standard MLM)
guaiol* norm QQ plot (standard MLM)
guaiol* norm (optimal MLMM)
gamma-eudesmol* norm (standard MLM)
gamma-eudesmol* norm QQ plot (standard MLM)
gamma-eudesmol* norm (optimal MLMM)
beta-eudesmol norm (standard MLM)
beta-eudesmol norm QQ plot (standard MLM)
beta-eudesmol norm (optimal MLMM)
−log10(p)
chromosome
Observed −log10(p)
Expected −log10(p)

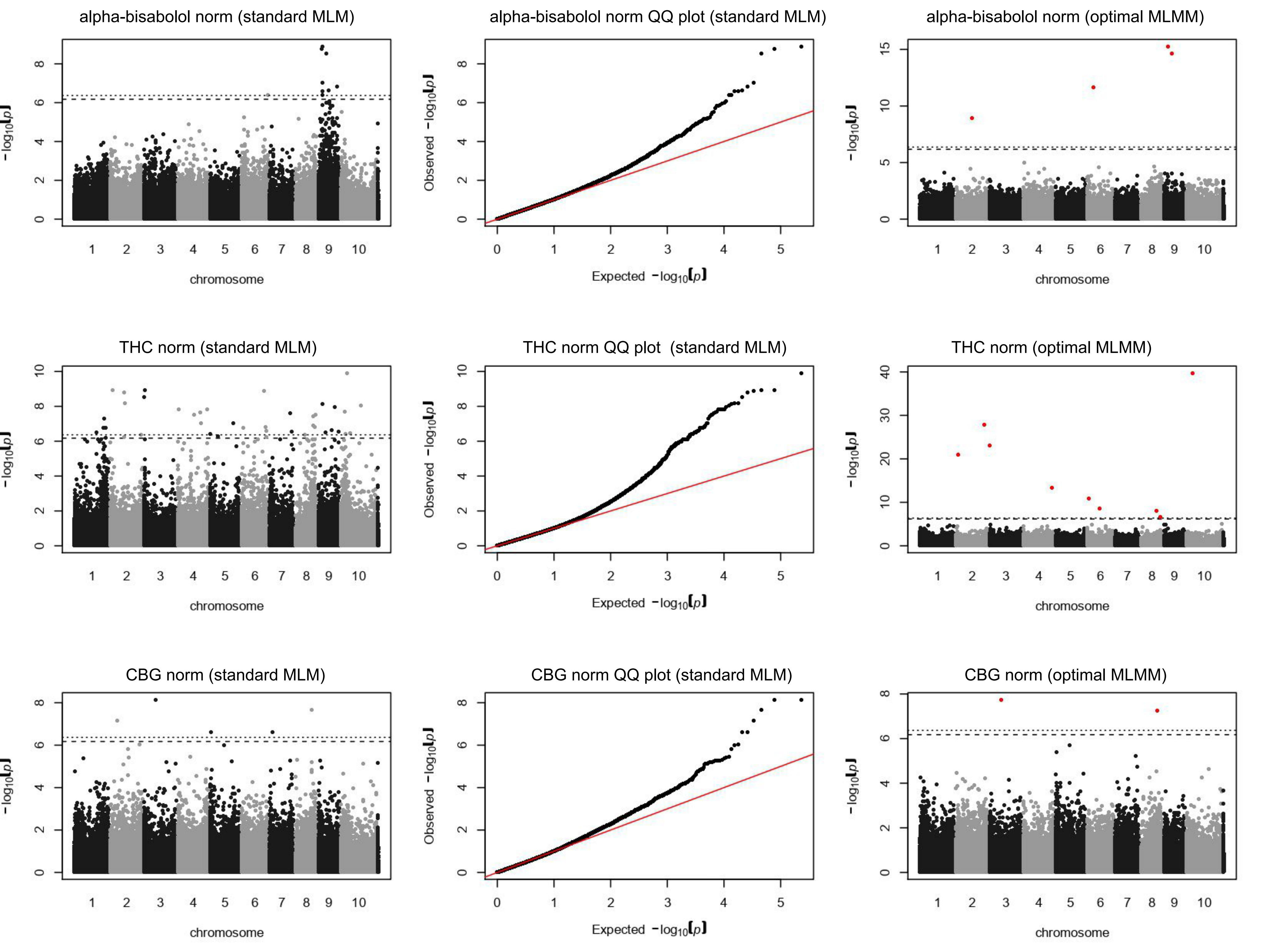

alpha-bisabolol norm (standard MLM)
alpha-bisabolol norm QQ plot (standard MLM)
alpha-bisabolol norm (optimal MLMM)
THC norm (standard MLM)
THC norm QQ plot (standard MLM)
THC norm (optimal MLMM)
CBG norm (standard MLM)
CBG norm QQ plot (standard MLM)
CBG norm (optimal MLMM)
chromosome
Expected −log10(p)
Observed −log10(p)
−log10(p)

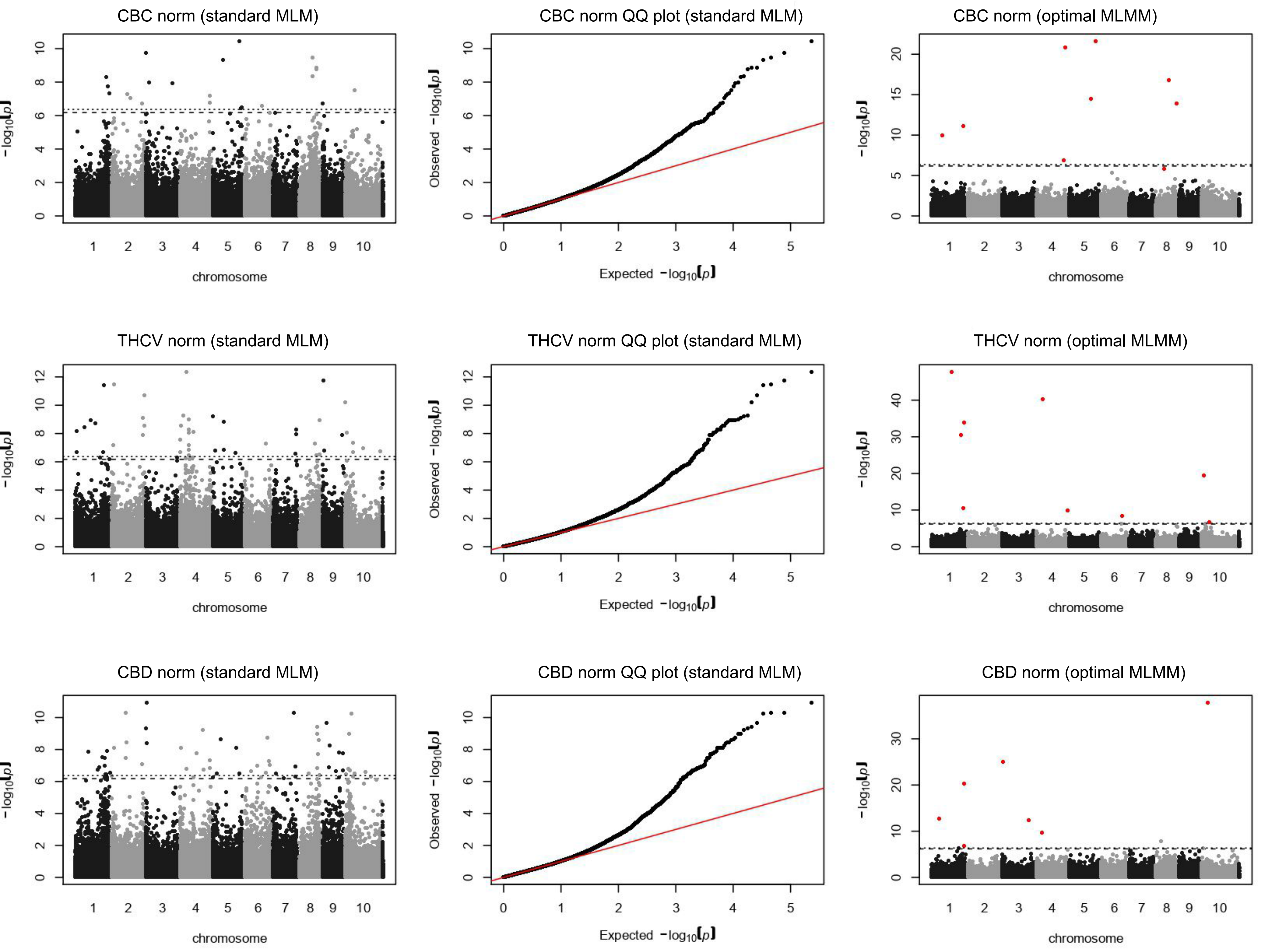

CBC norm (standard MLM)
CBC norm QQ plot (standard MLM)
CBC norm (optimal MLMM)
THCV norm (standard MLM)
THCV norm QQ plot (standard MLM)
THCV norm (optimal MLMM)
CBD norm (standard MLM)
CBD norm QQ plot (standard MLM)
CBD norm (optimal MLMM)
−log10(p)
Observed −log10(p)
Expected −log10(p)
chromosome

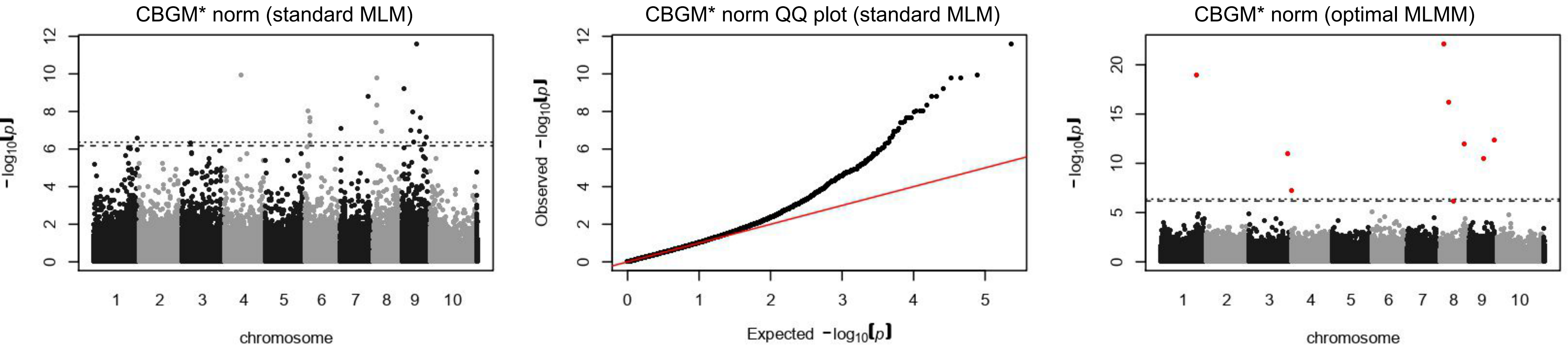

CBGM* norm (standard MLM)
CBGM* norm QQ plot (standard MLM)
CBGM* norm (optimal MLMM)
−log10(p)
Observed −log10(p)
Expected −log10(p)
chromosome

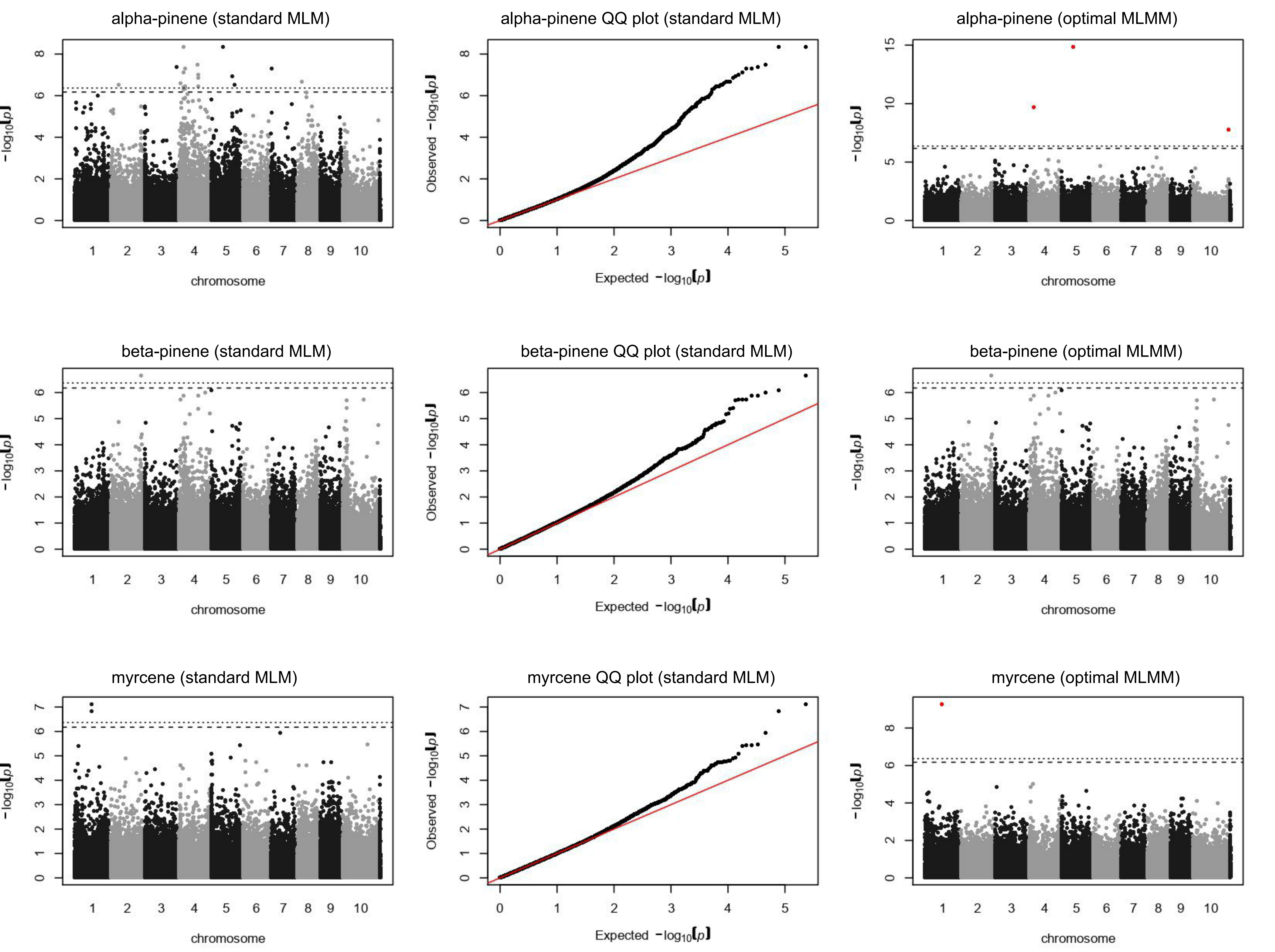
alpha-pinene (standard MLM)
alpha-pinene QQ plot (standard MLM)
alpha-pinene (optimal MLMM)
beta-pinene (standard MLM)
beta-pinene QQ plot (standard MLM)
beta-pinene (optimal MLMM)
myrcene (standard MLM)
myrcene QQ plot (standard MLM)
myrcene (optimal MLMM)
$-\log_{10}(p)$
Observed $-\log_{10}(p)$
Expected $-\log_{10}(p)$
chromosome
1 2 3 4 5 6 7 8 9 10

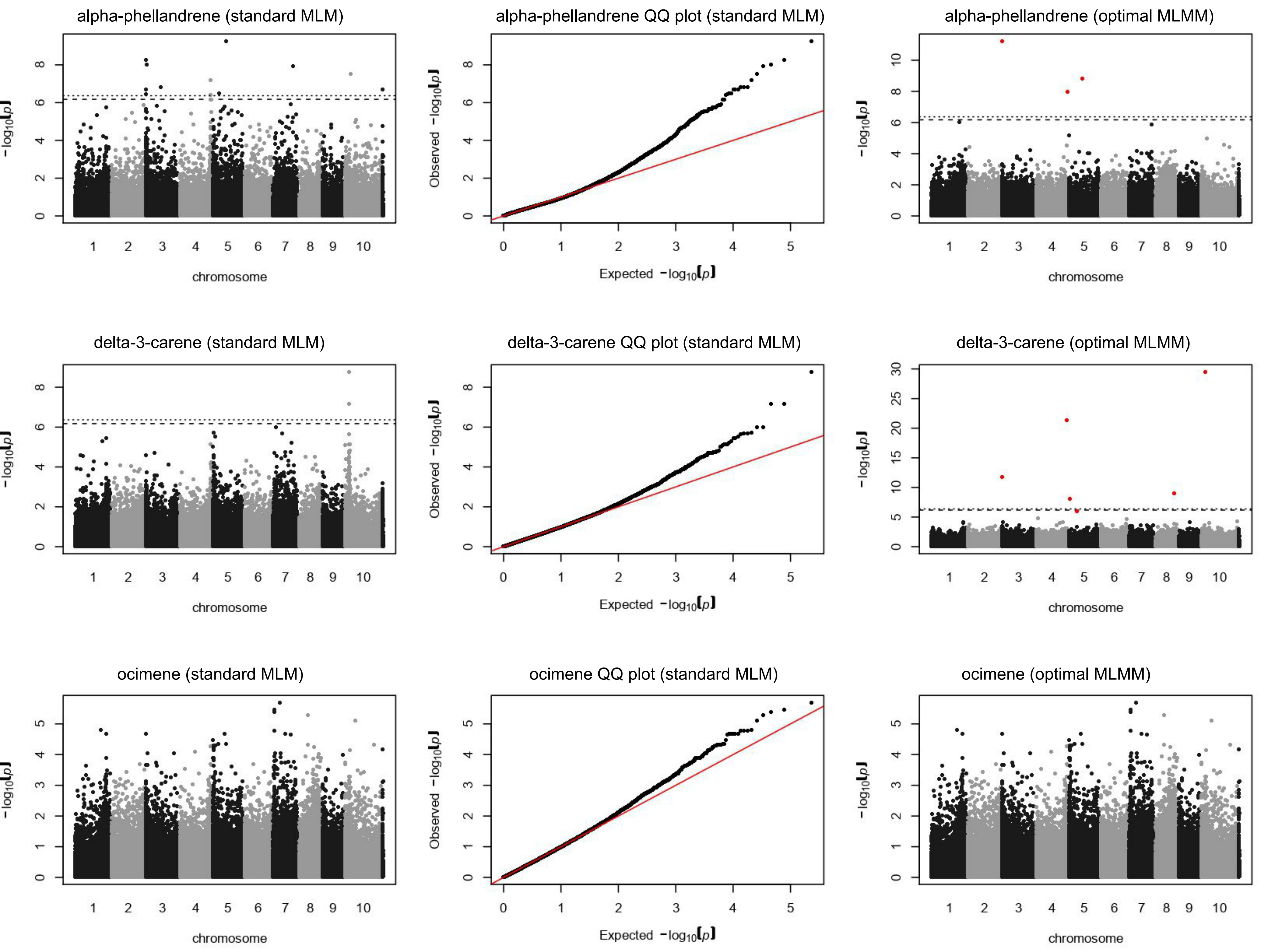

alpha-phellandrene (standard MLM)
alpha-phellandrene QQ plot (standard MLM)
alpha-phellandrene (optimal MLMM)
delta-3-carene (standard MLM)
delta-3-carene QQ plot (standard MLM)
delta-3-carene (optimal MLMM)
ocimene (standard MLM)
ocimene QQ plot (standard MLM)
ocimene (optimal MLMM)
chromosome
Expected −log10(p)
Observed −log10(p)
−log10(p)

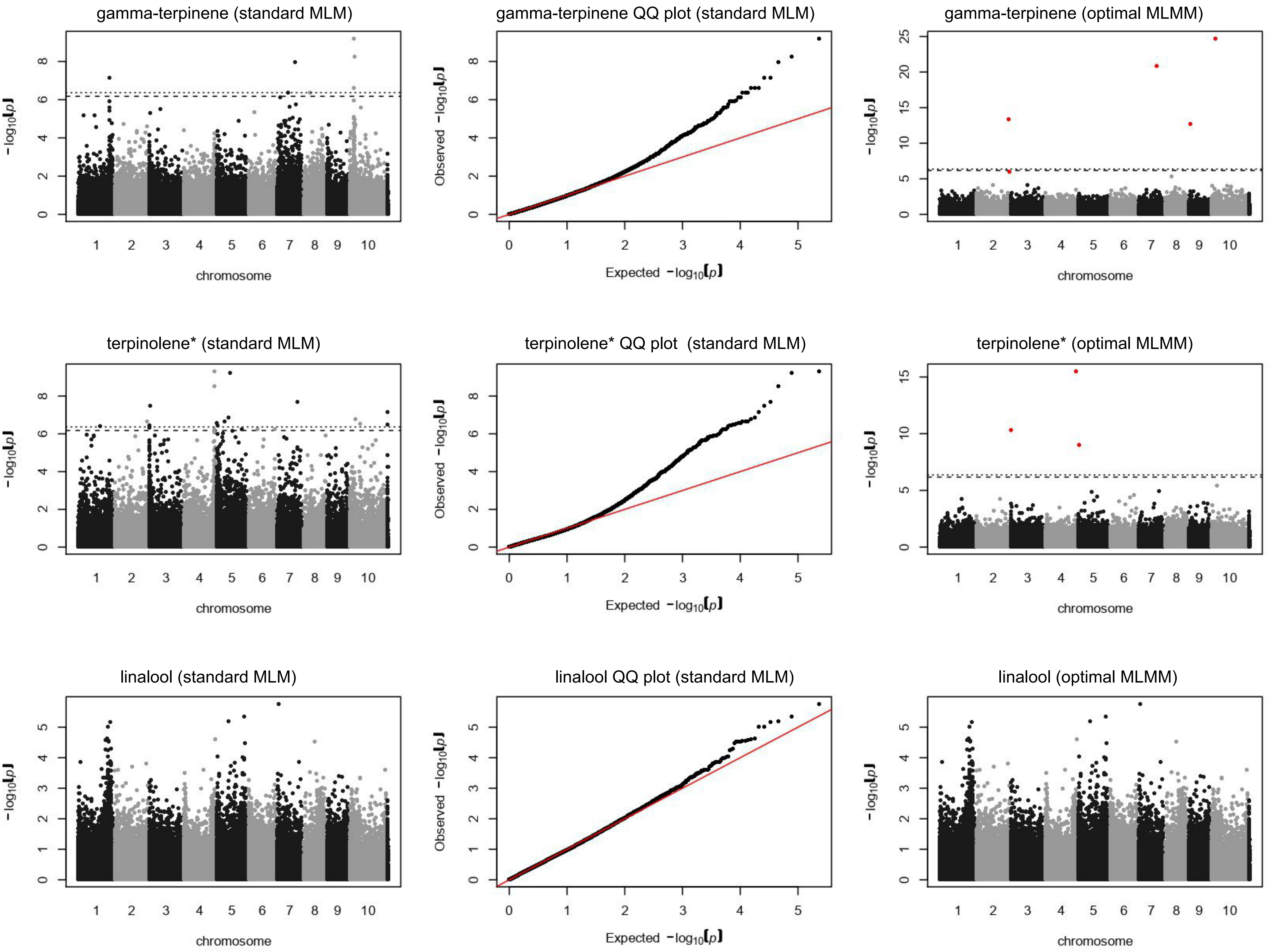
gamma-terpinene (standard MLM)
gamma-terpinene QQ plot (standard MLM)
gamma-terpinene (optimal MLMM)
terpinolene* (standard MLM)
terpinolene* QQ plot (standard MLM)
terpinolene* (optimal MLMM)
linalool (standard MLM)
linalool QQ plot (standard MLM)
linalool (optimal MLMM)
$-\log_{10}(p)$
Observed $-\log_{10}(p)$
Expected $-\log_{10}(p)$
chromosome
1 2 3 4 5 6 7 8 9 10

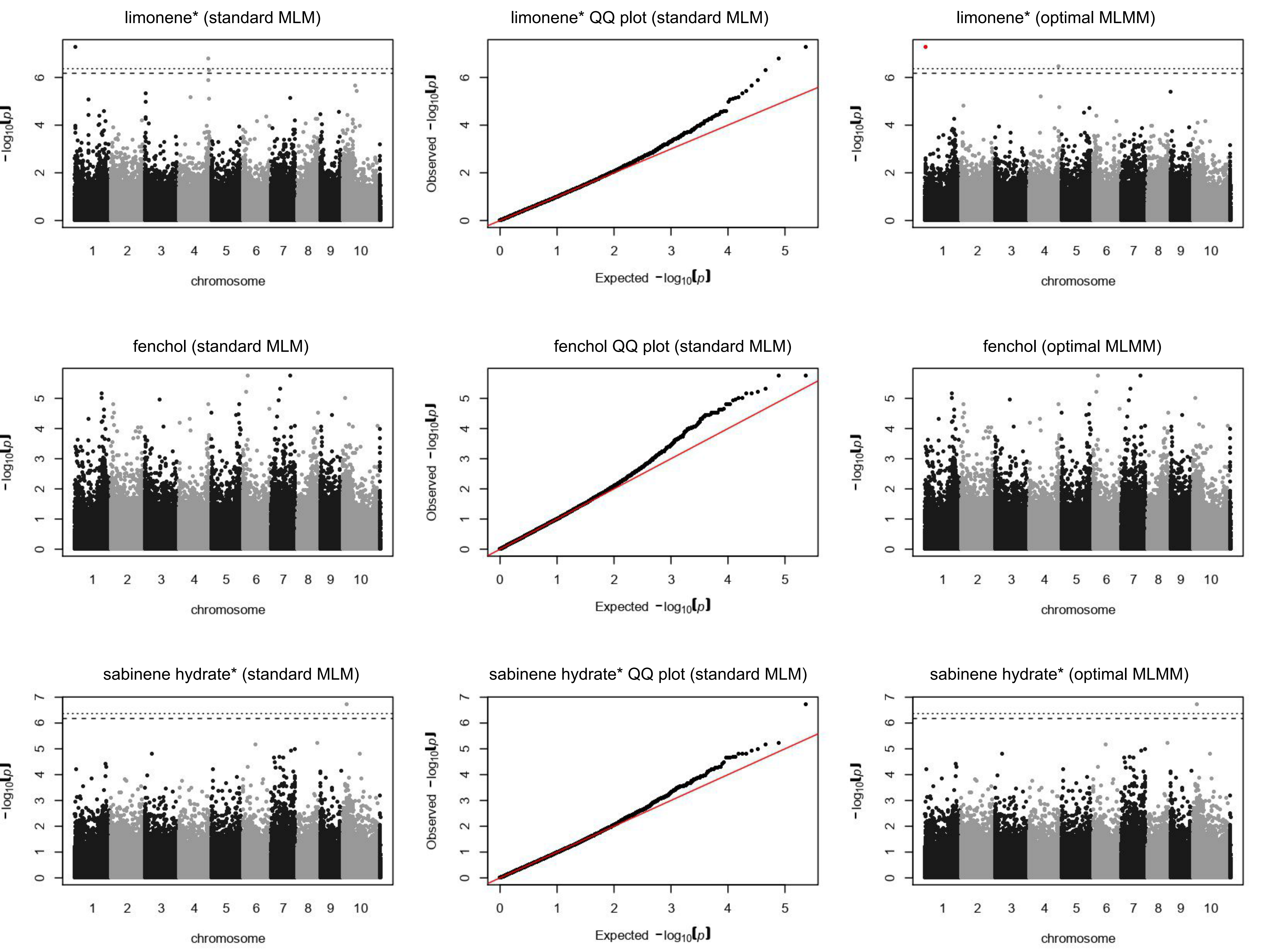

limonene* (standard MLM)
limonene* QQ plot (standard MLM)
limonene* (optimal MLMM)
fenchol (standard MLM)
fenchol QQ plot (standard MLM)
fenchol (optimal MLMM)
sabinene hydrate* (standard MLM)
sabinene hydrate* QQ plot (standard MLM)
sabinene hydrate* (optimal MLMM)
chromosome
Expected −log10(p)
Observed −log10(p)
−log10(p)

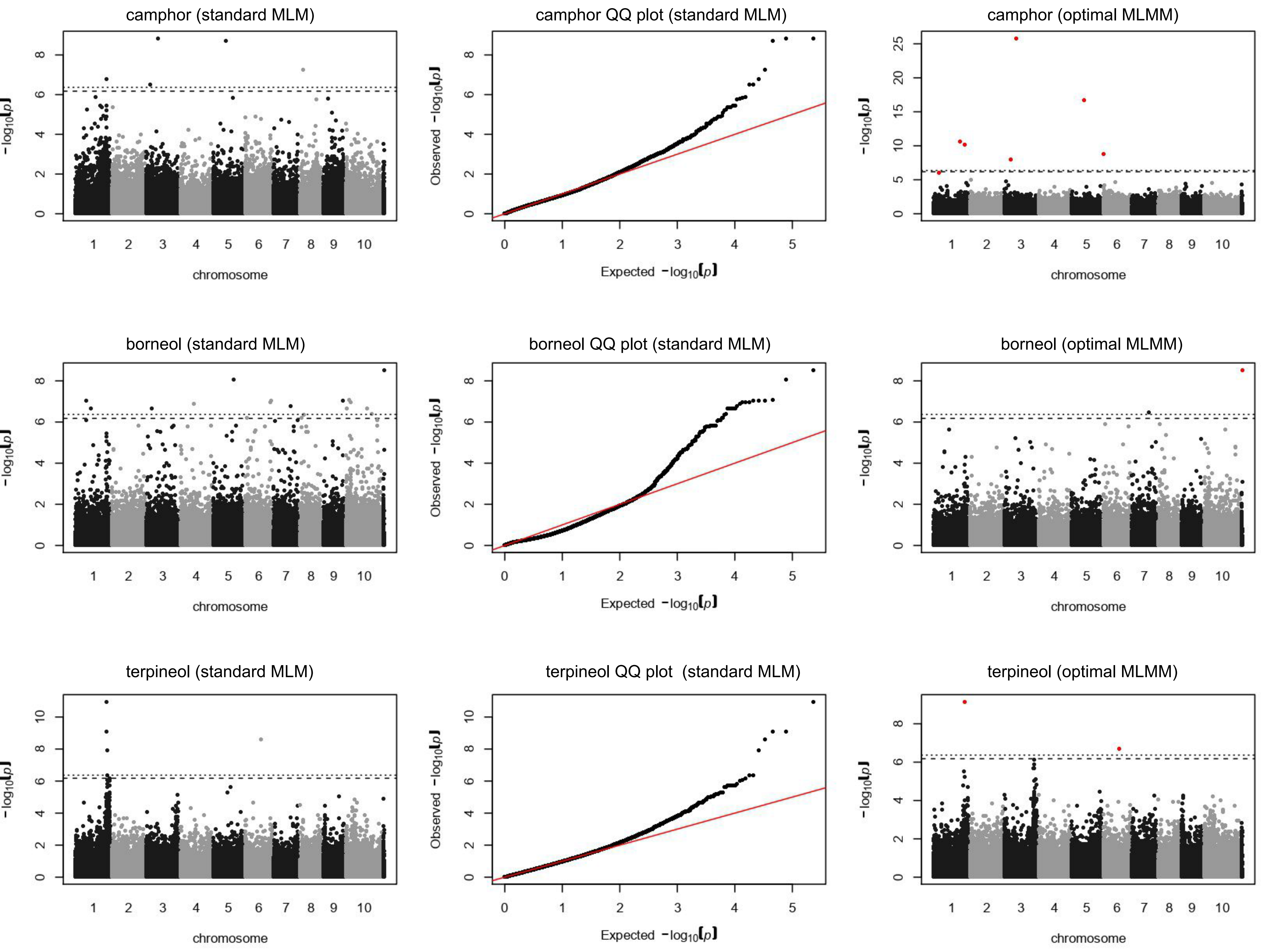

camphor (standard MLM)
camphor QQ plot (standard MLM)
camphor (optimal MLMM)
borneol (standard MLM)
borneol QQ plot (standard MLM)
borneol (optimal MLMM)
terpineol (standard MLM)
terpineol QQ plot (standard MLM)
terpineol (optimal MLMM)
chromosome
Observed −log10(p)
Expected −log10(p)
−log10(p)

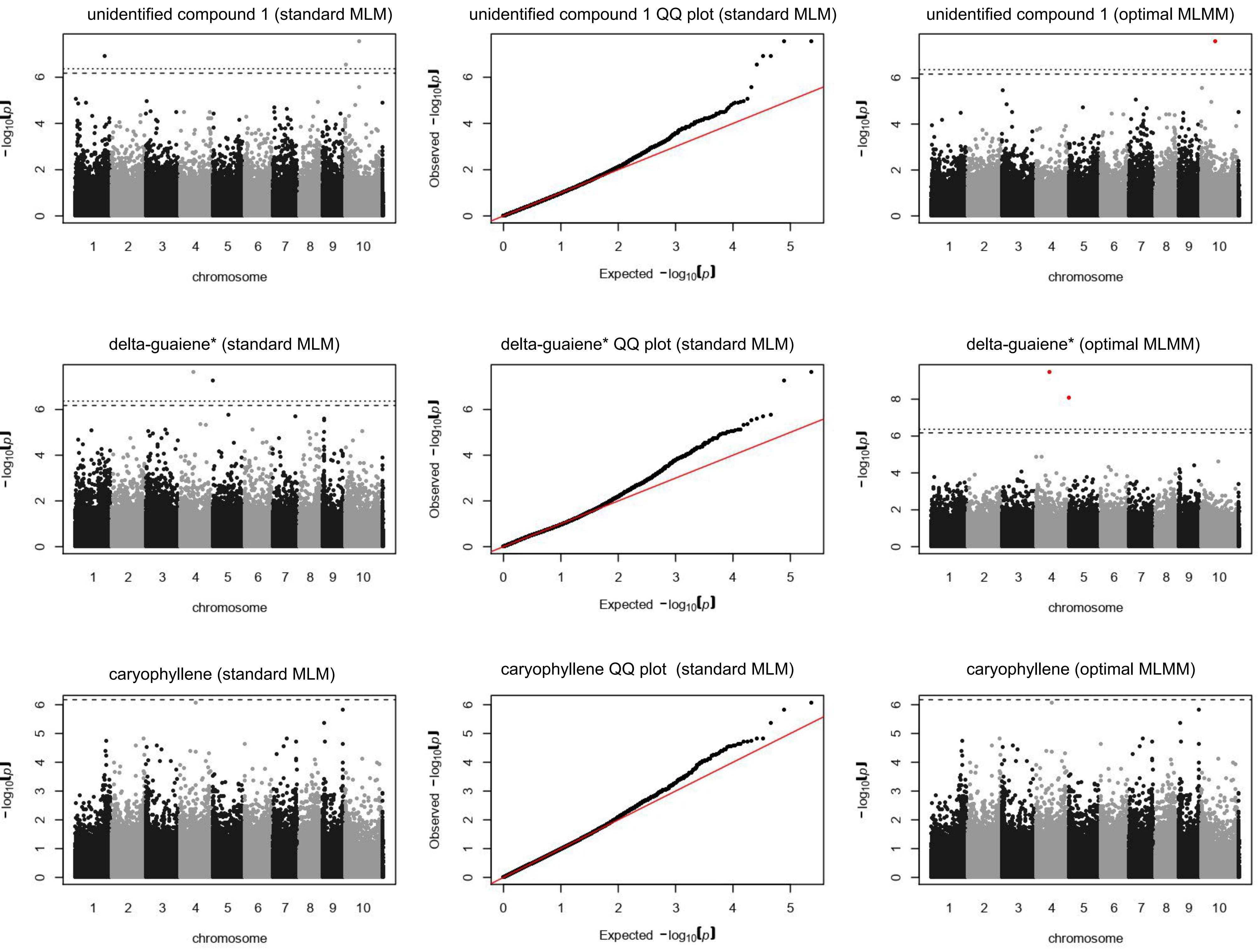
unidentified compound 1 (standard MLM)
unidentified compound 1 QQ plot (standard MLM)
unidentified compound 1 (optimal MLMM)
delta-guaiene* (standard MLM)
delta-guaiene* QQ plot (standard MLM)
delta-guaiene* (optimal MLMM)
caryophyllene (standard MLM)
caryophyllene QQ plot (standard MLM)
caryophyllene (optimal MLMM)
chromosome
Expected −log10(p)
Observed −log10(p)
−log10(p)

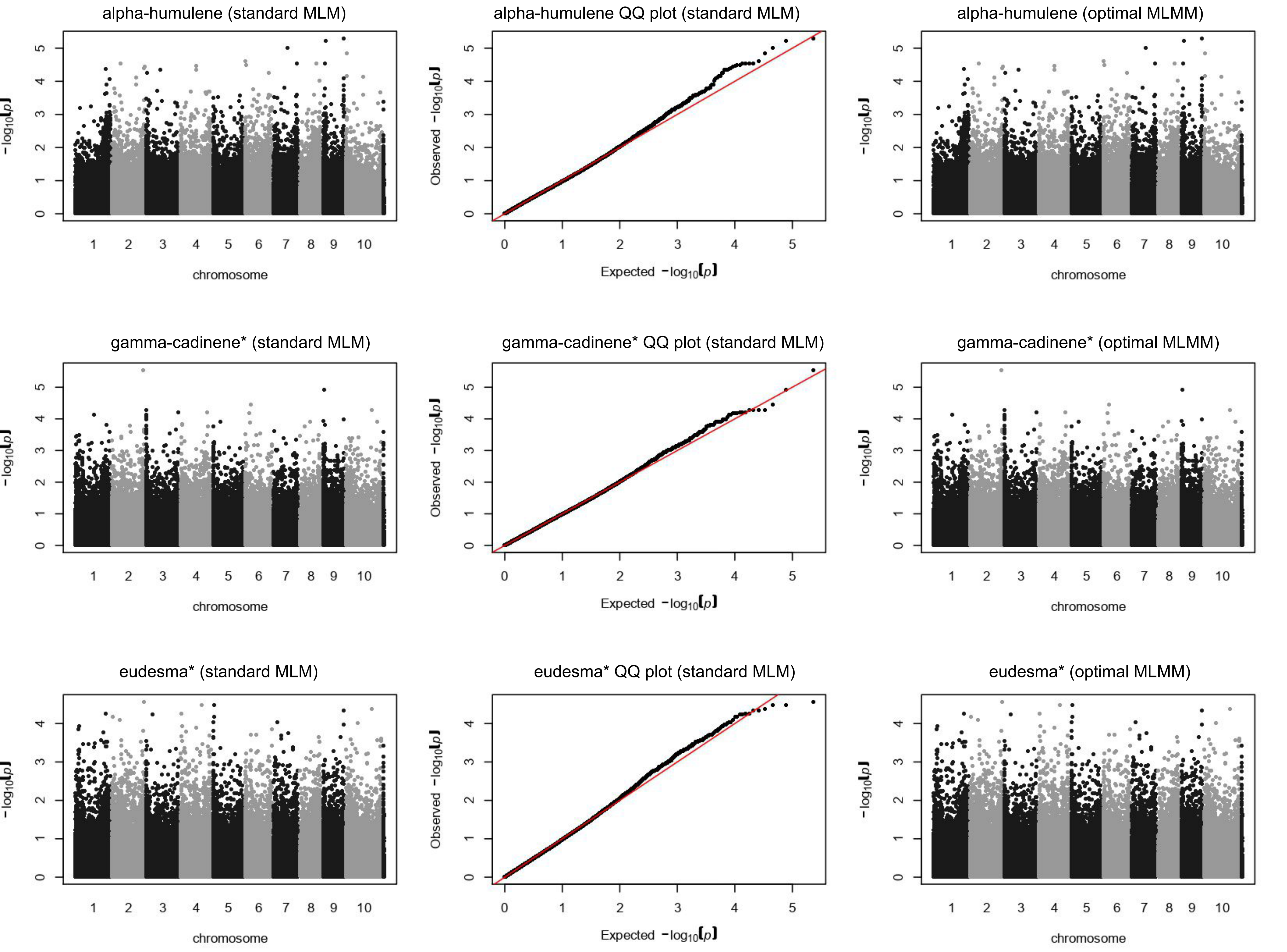

alpha-humulene (standard MLM)
alpha-humulene QQ plot (standard MLM)
alpha-humulene (optimal MLMM)
gamma-cadinene* (standard MLM)
gamma-cadinene* QQ plot (standard MLM)
gamma-cadinene* (optimal MLMM)
eudesma* (standard MLM)
eudesma* QQ plot (standard MLM)
eudesma* (optimal MLMM)
−log10(p)
Observed −log10(p)
Expected −log10(p)
chromosome

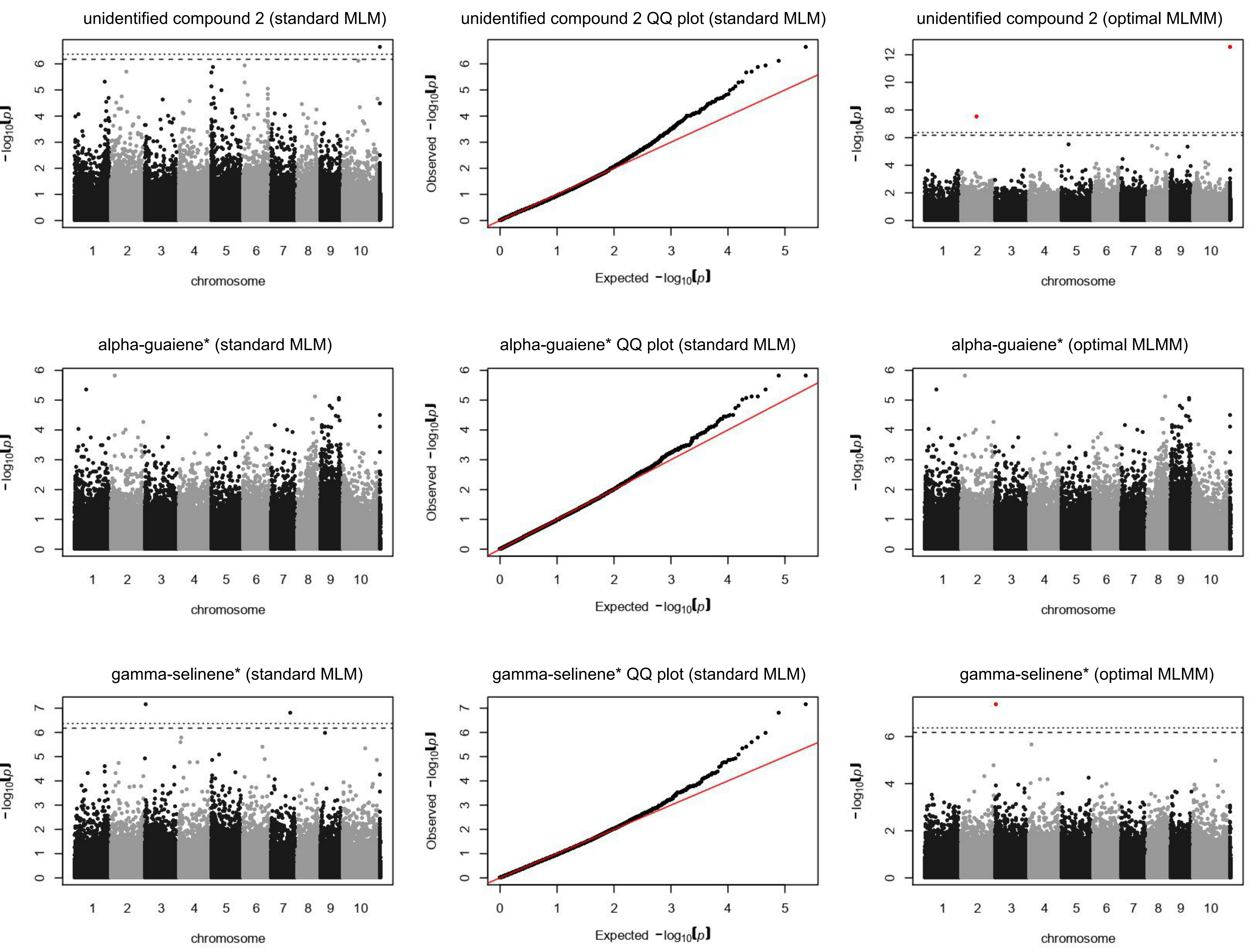

unidentified compound 2 (standard MLM)
unidentified compound 2 QQ plot (standard MLM)
unidentified compound 2 (optimal MLMM)
alpha-guaiene* (standard MLM)
alpha-guaiene* QQ plot (standard MLM)
alpha-guaiene* (optimal MLMM)
gamma-selinene* (standard MLM)
gamma-selinene* QQ plot (standard MLM)
gamma-selinene* (optimal MLMM)
$-\log_{10}(p)$
Observed $-\log_{10}(p)$
Expected $-\log_{10}(p)$
chromosome

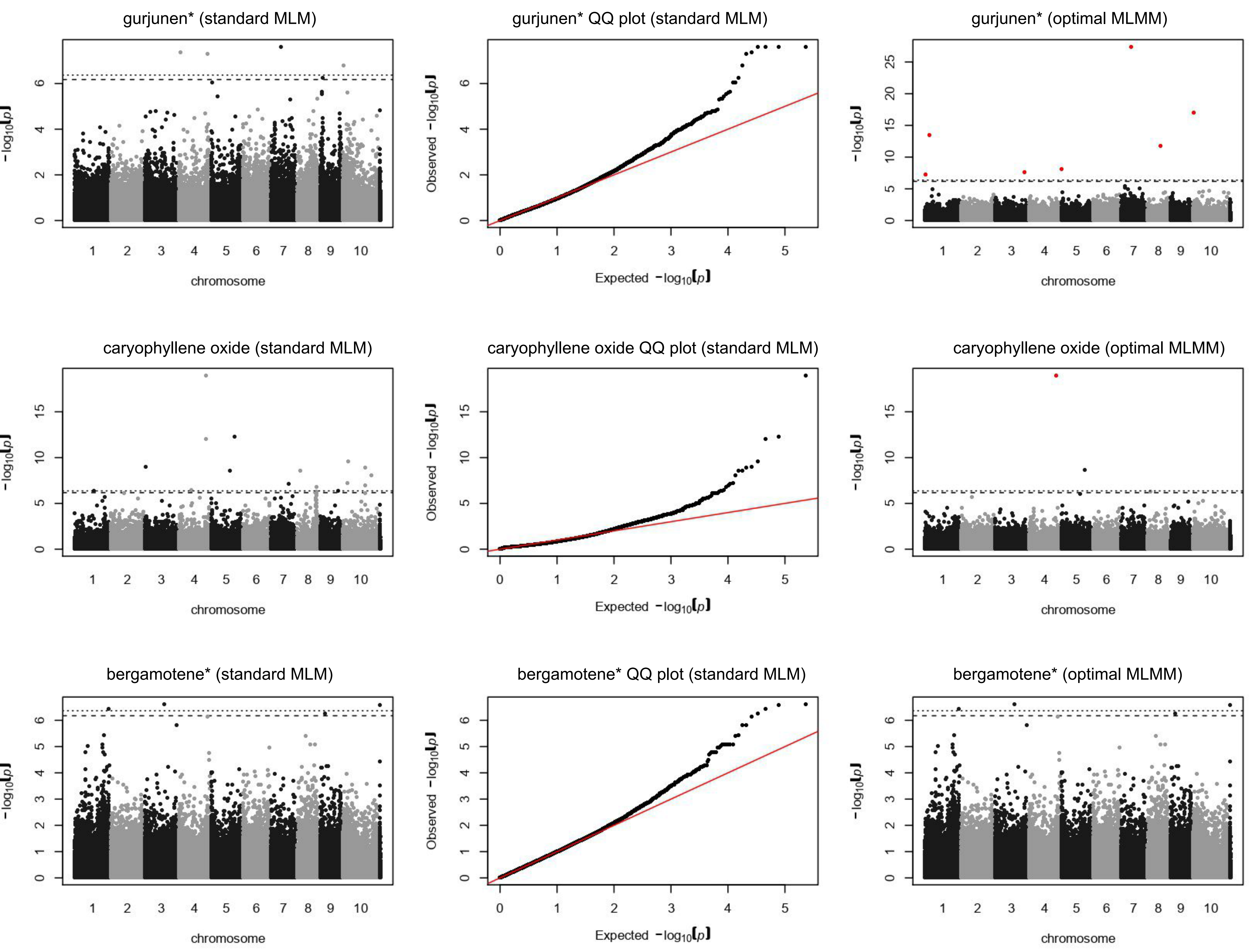
gurjunen* (standard MLM)
gurjunen* QQ plot (standard MLM)
gurjunen* (optimal MLMM)
caryophyllene oxide (standard MLM)
caryophyllene oxide QQ plot (standard MLM)
caryophyllene oxide (optimal MLMM)
bergamotene* (standard MLM)
bergamotene* QQ plot (standard MLM)
bergamotene* (optimal MLMM)
chromosome
Expected −log10(p)
Observed −log10(p)
−log10(p)

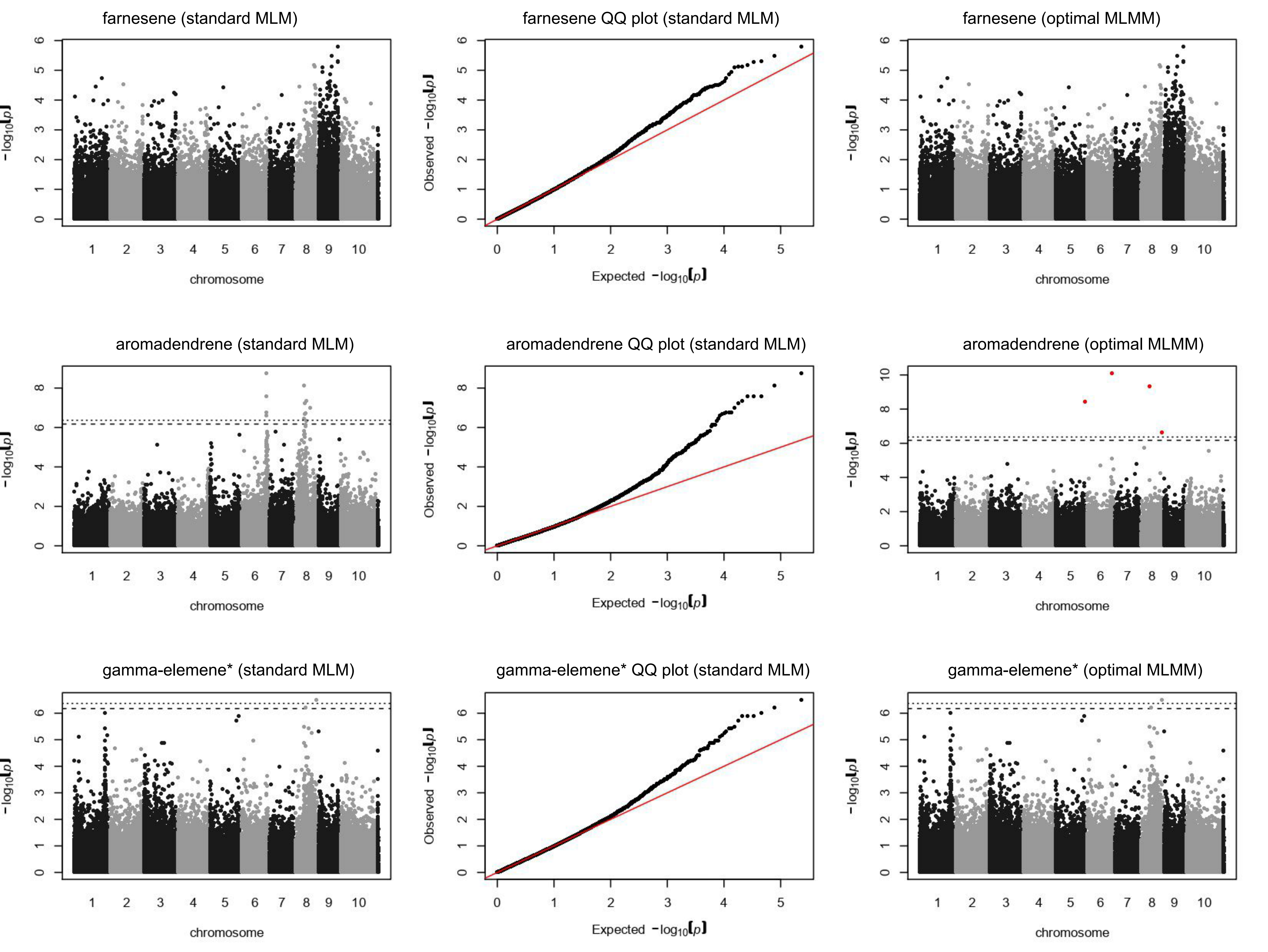
farnesene (standard MLM)
farnesene QQ plot (standard MLM)
farnesene (optimal MLMM)
aromadendrene (standard MLM)
aromadendrene QQ plot (standard MLM)
aromadendrene (optimal MLMM)
gamma-elemene* (standard MLM)
gamma-elemene* QQ plot (standard MLM)
gamma-elemene* (optimal MLMM)
−log10(p)
chromosome
1 2 3 4 5 6 7 8 9 10
Observed −log10(p)
Expected −log10(p)

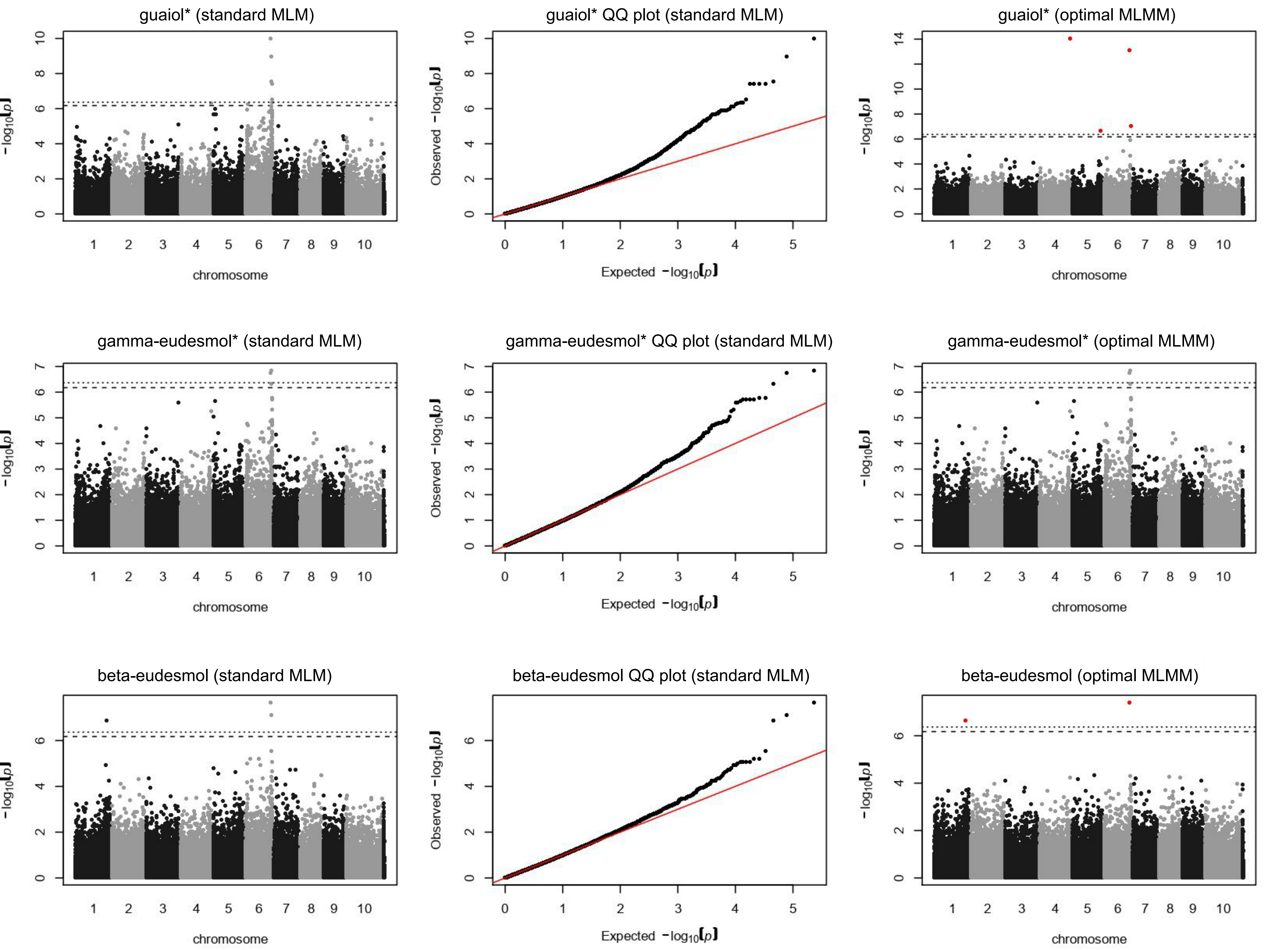
guaiol* (standard MLM)
guaiol* QQ plot (standard MLM)
guaiol* (optimal MLMM)
gamma-eudesmol* (standard MLM)
gamma-eudesmol* QQ plot (standard MLM)
gamma-eudesmol* (optimal MLMM)
beta-eudesmol (standard MLM)
beta-eudesmol QQ plot (standard MLM)
beta-eudesmol (optimal MLMM)
$-\log_{10}(p)$
Observed $-\log_{10}(p)$
Expected $-\log_{10}(p)$
chromosome
1 2 3 4 5 6 7 8 9 10

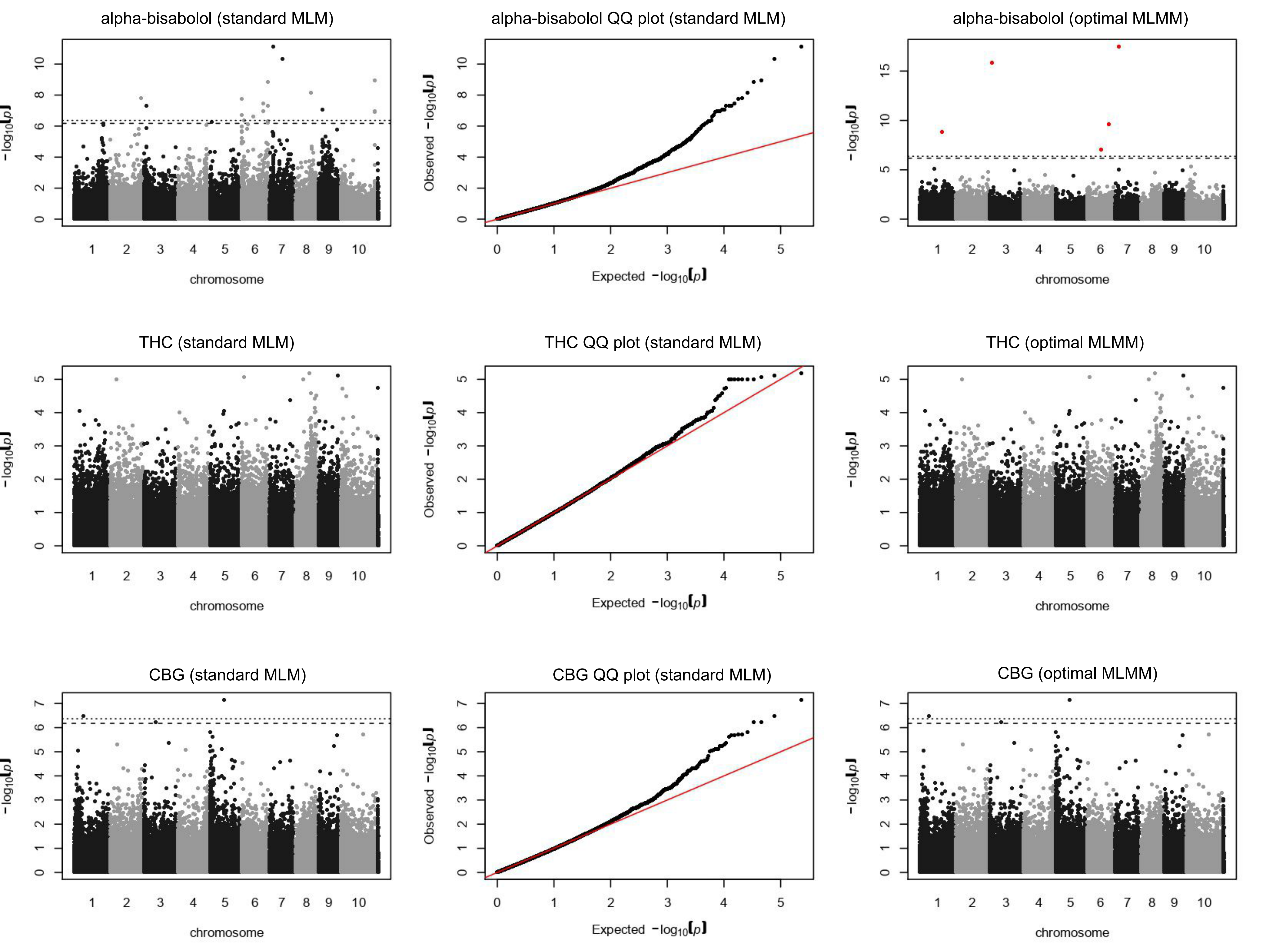
alpha-bisabolol (standard MLM)
alpha-bisabolol QQ plot (standard MLM)
alpha-bisabolol (optimal MLMM)
THC (standard MLM)
THC QQ plot (standard MLM)
THC (optimal MLMM)
CBG (standard MLM)
CBG QQ plot (standard MLM)
CBG (optimal MLMM)
$-\log_{10}(p)$
chromosome
1 2 3 4 5 6 7 8 9 10
Observed $-\log_{10}(p)$
Expected $-\log_{10}(p)$

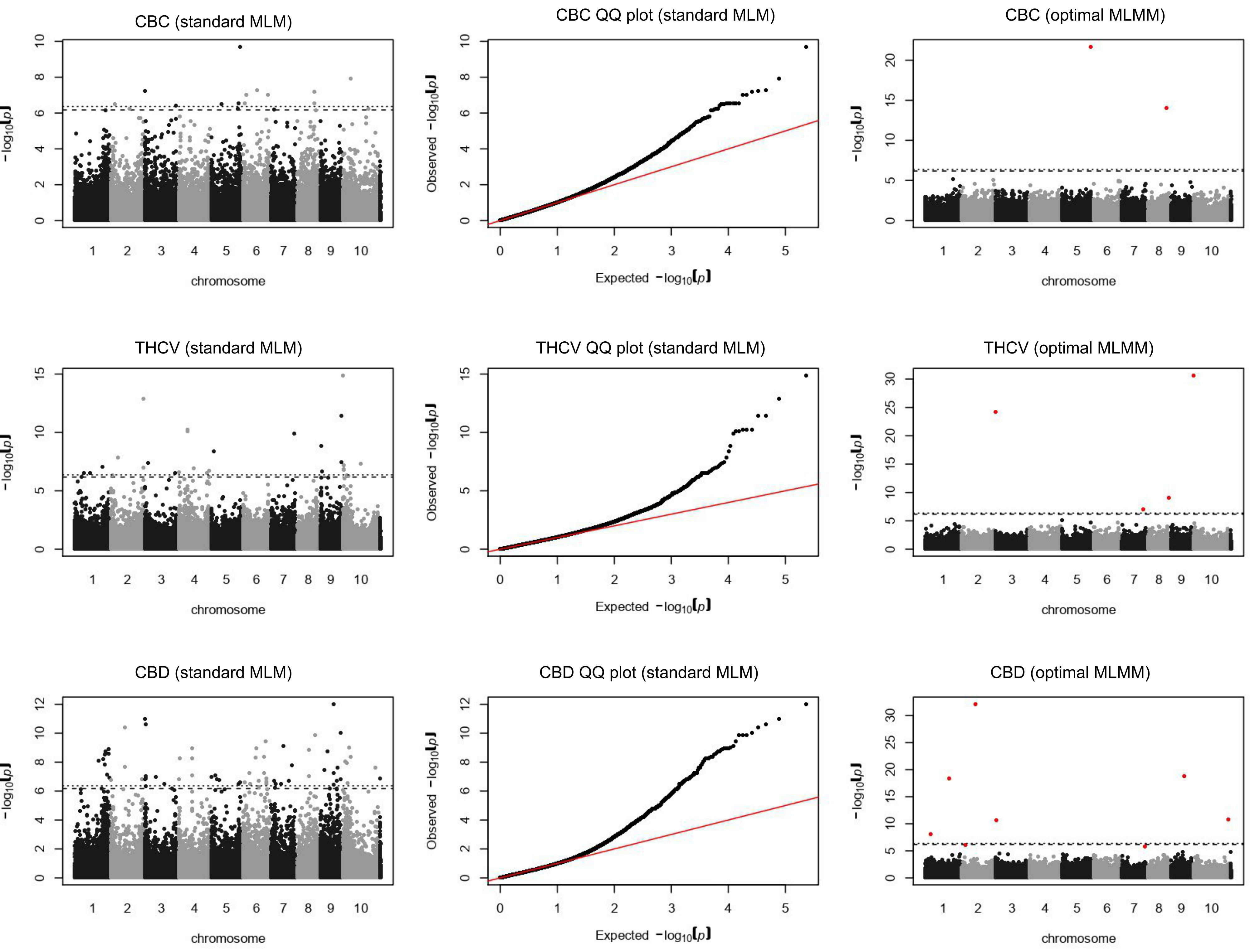

CBC (standard MLM)
CBC QQ plot (standard MLM)
CBC (optimal MLMM)
THCV (standard MLM)
THCV QQ plot (standard MLM)
THCV (optimal MLMM)
CBD (standard MLM)
CBD QQ plot (standard MLM)
CBD (optimal MLMM)
chromosome
Expected −log10(p)
Observed −log10(p)
−log10(p)

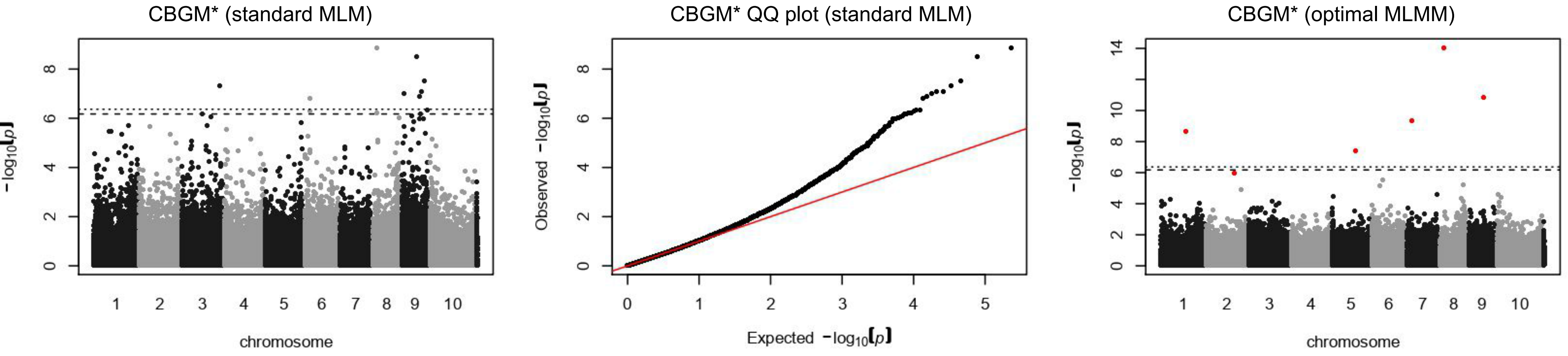

CBGM* (standard MLM)
CBGM* QQ plot (standard MLM)
CBGM* (optimal MLMM)
$-\log_{10}(p)$
chromosome
Observed $-\log_{10}(p)$
Expected $-\log_{10}(p)$
1 2 3 4 5 6 7 8 9 10
